# On‐Off Phagocytosis and Switchable Macrophage Activation Stimulated with NIR for Infected Percutaneous Tissue Repair of Polypyrrole‐Coated Sulfonated PEEK

**DOI:** 10.1002/advs.202205048

**Published:** 2022-12-14

**Authors:** Xingdan Liu, Haifeng Zhang, Bangcheng Yan, Kelvin W. K. Yeung, Yun Liao, Liping Ouyang, Xuanyong Liu

**Affiliations:** ^1^ State Key Laboratory of High Performance Ceramics and Superfine Microstructure Shanghai Institute of Ceramics Chinese Academy of Sciences Shanghai 200050 China; ^2^ Center of Materials Science and Optoelectronics Engineering University of Chinese Academy of Sciences Beijing 100049 China; ^3^ Department of Pharmacy Tongren Hospital Shanghai Jiao Tong University School of Medicine Shanghai 200336 China; ^4^ Hongqiao International Institute of Medicine Shanghai Jiao Tong University School of Medicine Shanghai 200336 China; ^5^ Shenzhen Key Laboratory for Innovative Technology in Orthopaedic Trauma Guangdong Engineering Technology Research Center for Orthopaedic Trauma Repair Department of Orthopaedics and Traumatology The University of Hong Kong Shenzhen Hospital Shenzhen 518053 China; ^6^ School of Chemistry and Materials Science Hangzhou Institute for Advanced Study University of Chinese Academy of Sciences 1 Sub‐lane Xiangshan Hangzhou 310024 China

**Keywords:** complement, macrophage activation, on‐off phagocytosis, polyether ether ketone

## Abstract

Intelligent control of the immune response is essential for obtaining percutaneous implants with good sterilization and tissue repair abilities. In this study, polypyrrole (Ppy) nanoparticles enveloping a 3D frame of sulfonated polyether ether ketone (SP) surface are constructed, which enhance the surface modulus and hardness of the sulfonated layer by forming a cooperative structure of simulated reinforced concrete and exhibit a superior photothermal effect. Ppy‐coated SP could quickly accumulate heat on the surface by responding to 808 nm near‐infrared (NIR) light, thereby killing bacteria, and destroying biofilms. Under NIR stimulation, the phagocytosis and M1 activation of macrophages cultured on Ppy‐coated SP are enhanced by activating complement 3 and its receptor, CD11b. Phagocytosis and M1 activation are impaired along with abolishment of NIR stimulation in the Ppy‐coated SP group, which is favorable for tissue repair. Ppy‐coated SP promotes Collagen‐I, vascular endothelial growth factor, connective tissue growth factor, and *α*‐actin (Acta2) expression by inducing M2 polarization owing to its higher surface modulus. Overall, Ppy‐coated SP with enhanced mechanical properties could be a good candidate for clinical percutaneous implants through on‐off phagocytosis and switchable macrophage activation stimulated with NIR.

## Graphical abstract

Polypyrrole (Ppy) nanoparticles enveloping a porous skeleton of sulfonated polyether ether ketone (PEEK) enhance the surface modulus and hardness of sulfonated layer. Ppy‐coated sulfonated PEEK improves phagocytosis and M1 macrophage activation through turning on near‐infrared (NIR) to help clear bacteria; and suppressed phagocytosis and a switch to M2 macrophage occur with closed NIR, which is facilitated to tissue repair.

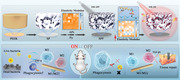

## 1 Introduction

As diseases, traffic accidents, and the aging population increase, the demand for percutaneous implants is rapidly increasing. Polyether ether ketone (PEEK) is often used as a replacement material for bone, dental implants, and surgical instruments in interventional therapy owing to its good biocompatibility, mechanical properties, and chemical stability.^[^ ^1^, ^2^ ^]^ However, PEEK is bioinert and has no antibacterial ability, which causes poor biological bonding between the implant and tissue, potentially leading to surgical failure.^[^ ^3^, ^4^, ^5^, ^6^ ^]^ In recent decades, sulfonation has been applied to endow PEEK with a porous structure and has been widely verified to have good sterilization and tissue repair abilities.^[^ ^7^, ^8^ ^]^ However, the impaired mechanical properties of the sulfonated layer remain the biggest obstacle to the clinical application of sulfonated PEEK (SP). Therefore, the mechanical properties of SP should be improved while obtaining superior sterilization and tissue repair properties.

The success of percutaneous implant relies on resistance to bacterial infection and biological healing of the implant surface with the soft tissue.^[^ ^9^, ^10^ ^]^ Percutaneous implants have a high risk of infection owing to their relatively open and complex service environments. In particular, biofilm‐associated implant infections account for ≈65% of all bacterial infections in healthcare settings. The formation of biofilms renders some antibacterial materials ineffective and inhibits host immunity and antibiotic penetration by the biofilm matrix, thereby prolonging the treatment period.^[^ ^11^, ^12^ ^]^ Therefore, SP materials with superior antibacterial properties must be developed for percutaneous implantation therapy in clinical practice. Photothermal therapy (PTT) has recently attracted remarkable attention in the antibacterial field owing to its high spatiotemporal selectivity, low invasiveness, and recurrence probability.^[^ ^13^, ^14^, ^15^ ^]^ In particular, near‐infrared (NIR) light‐mediated PTT is considered a universal and effective antimicrobial therapy.^[^ ^16^, ^17^, ^18^ ^]^ Several PTT reagents can respond to NIR, including inorganic materials, metals, and polymers. Li et al. developed a biocompatible phototherapeutic system comprising MoS_2_, IR780 photosensitizer, and arginine‐glycine‐aspartic acid‐cysteine to eradicate biofilms on titanium implants using NIR‐mediated PTT and photodynamic therapy.^[^ ^15^ ^]^ Hu et al. prepared an antibacterial material by forming a rod‐shaped core–shell‐shell Au‐Ag‐Au nanoheater in the NIR region.^[^ ^19^ ^]^ Yan et al. grafted polyaniline (PANI) and ethylene glycol chitosan onto the surface of persistent luminescent nanoparticles to develop an intelligent platform that displayed significant therapeutic effects on bacterial infection abscesses.^[^ ^20^ ^]^

PTT can effectively sterilize bacteria on the surface of implants; however, it is difficult to kill dissociative bacteria in the tissue. Intelligent control of the immune response is an emerging selection to obtain percutaneous implants with good sterilization and tissue repair abilities.^[^ ^11^, ^21^, ^22^ ^]^ Macrophages are important immune cells that play an essential role in innate immune defense and specific immune activation against bacteria.^[^ ^23^, ^24^, ^25^ ^]^ Macrophages can recognize bacteria via cytoplasmic pattern recognition receptors (PRRs). Surface PRRs, such as C‐type lectins and mannose receptors (MR), recognize and bind bacteria to initiate phagocytosis. Moreover, some non‐cellular molecules in the defense system, such as complement, can recognize, and modify bacterial surfaces to promote macrophages to indirectly recognize and bind the invading bacteria through the receptors of these molecules. Soluble PRRs, including complement components C1q, C3, C3b, mannose‐binding lectin (MBL), and surfactant proteins, can promote the opsonization of bacteria. Notably, opsonized bacteria can bind to C receptors (C1qR, CR3, and CR4). Further, downstream signaling cascades associated with these receptors induce receptor‐mediated phagocytosis.^[^ ^21^, ^26^, ^27^, ^28^ ^]^ On the other hand, macrophages can be activated to the pro‐inflammatory M1 type or pro‐healing M2 type. M1 mainly induces different pro‐inflammatory responses by expressing and/or secreting chemokines and cytokines and releasing antimicrobial effectors.^[^ ^29^, ^30^ ^]^ During bacterial invasion, M1 can secrete chemokines, such as monocyte chemoattractant protein‐1 (MCP‐1), to attract more macrophages and immune cells to the site of infection. M1 can also secrete pro‐inflammatory factors, including tumor necrosis factor‐*α* (TNF‐*α*), interleukin‐1*β* (IL‐1*β*), IL‐6, and IL‐23, to eliminate bacteria and maintain inflammation. M1 can release antimicrobial peptides, active substances, and other antibacterial molecules to kill bacteria.^[^ ^31^, ^32^, ^33^ ^]^

In the late stages, tissue repair is another important issue after controlling for bacterial infection. As the removal of invading bacteria reaches the healing stage, M2 polarization occurs through the bypass activation pathway. M2 macrophages express key cytokines and growth factors to promote cell proliferation, differentiation, extracellular matrix (ECM) deposition, and angiogenesis to facilitate tissue healing.^[^ ^34^, ^35^ ^]^ For example, paracrine factors, such as IL‐4, IL‐10, and transforming growth factor‐*β* (TGF‐*β*) secreted by M2, can stimulate the action of non‐immune cells, such as fibroblast proliferation, collagen secretion, and gene expression, including connective tissue growth factor (CTGF), to promote wound contraction and remodeling.^[^ ^36^, ^37^ ^]^ M2 also facilitates the vascularization of endothelial cells by secreting vascular endothelial growth factor (VEGF).^[^ ^38^, ^39^, ^40^ ^]^ Therefore, intelligent regulation of macrophage immune function to match the needs of different scenarios is key to implant success.

**Scheme 1:**
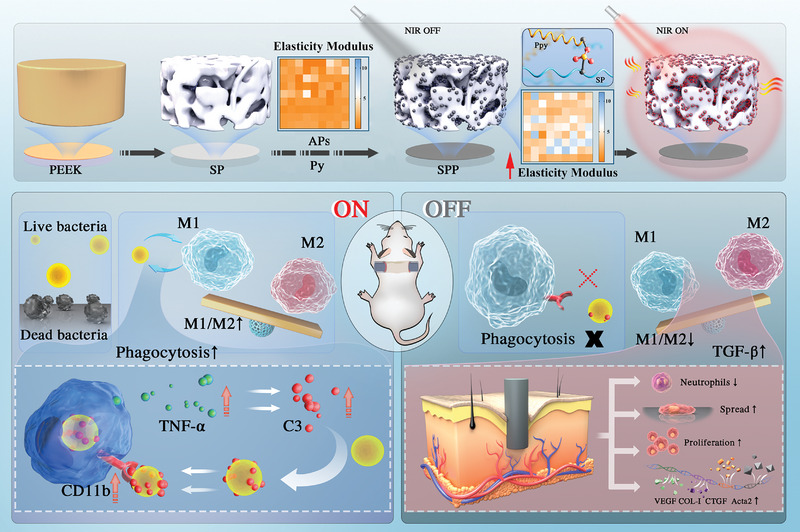
Schematic illustration of PEEK modification process through polypyrrole (Ppy). Ppy caused in situ confinement effect in the 3D skeleton structure of sulfonated PEEK (SP) by forming covalent bond, and it improved the mechanical properties of the sulfonated layer. Ppy coated SP (SPP) had superior photothermal effect in response to NIR. With NIR on, Ppy coated SP could kill bacteria cultured on surface; and enhance phagocytosis and M1 activation of macrophage cultured on its surface through activating C3 and its receptor, CD11b, thereby enhancing the removal of dissociative bacteria. As NIR removal, complement‐mediated phagocytosis weakened and macrophages switched to M2 due to higher modulus of SPP, which reduced inflammation and promoted collagen secretion, blood vessel formation, thereby accelerating tissue healing. Py: pyrrole; APS: Ammonium persulfate. TNF‐*α*: Tumor necrosis factor‐*α*; C3: Complement 3; CD11b: Receptor of C3; VEGF: Vascular endothelial growth factor; COL‐I: Collagen‐I; CTGF: Connective tissue growth factor; Acta2: *α*‐actin.

Polypyrrole (Ppy) is a good choice for NIR‐response reagents. Ppy has been applied in the field of biomedicine owing to its good biocompatibility, stability, electrochemical switching, and immunoregulation.^[^ ^41^, ^42^ ^]^ Consequently, Ppy nanoparticles have been generated via in situ polymerization in the 3D porous structure domain of SP to wrap the pore skeleton and effectively exert a photothermal antibacterial effect, as depicted in **Scheme** **1**. The mechanism of the on‐off photothermal response in coordination with the immune system for the killing of bacteria and promotion of tissue healing was investigated in this study.

## 2 Results

### 2.1 Surface Characterization of Ppy‐Coated SP

**Figure 1:**
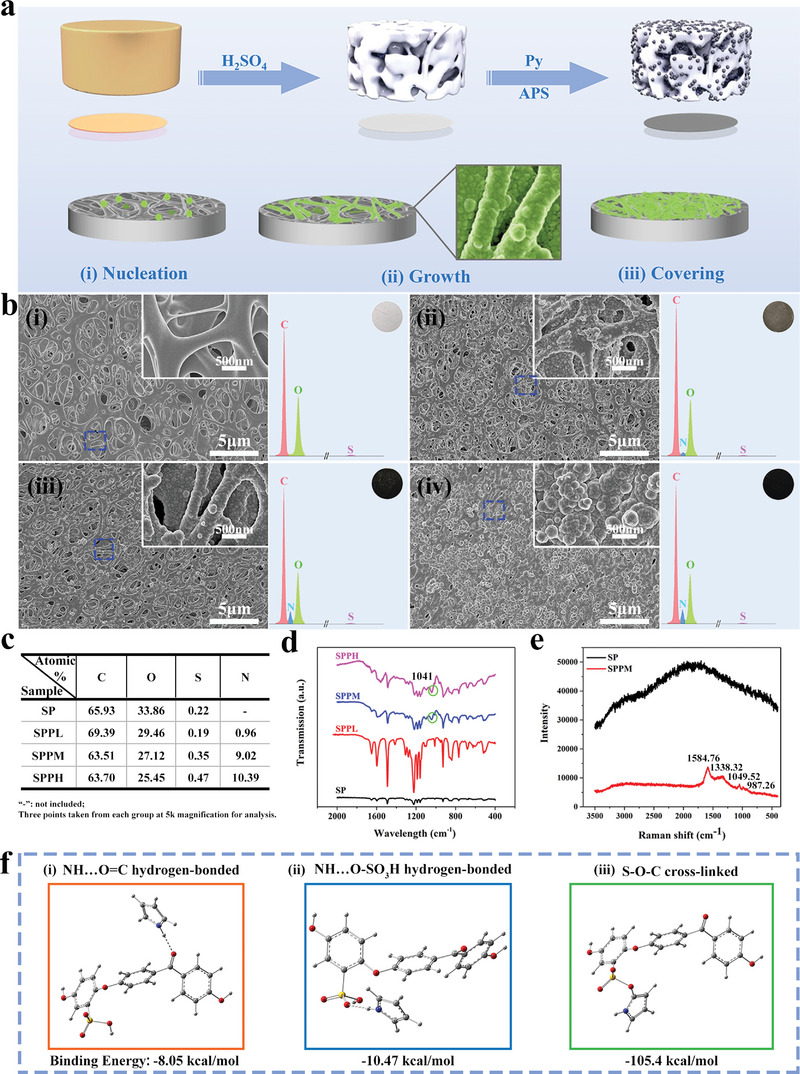
Surface morphologies and characterization. a) Schematic diagram of the sulfonated PEEK coated with Ppy. b) Surface morphologies, elemental composition, and macroscopic photos of i) SP, ii) SPPL, iii) SPPM, and iv) SPPH. The inset on top right corner is high magnification of dash line marked area, respectively. c) Surface element content of each group. d) FTIR spectra. e) Raman spectroscopy. f) Simulation calculation of the bonding energies between pyrrole monomer and sulfonated PEEK substrate. i) Hydrogen bonds with ketone groups on the substrate; ii) Hydrogen bonds with sulfonic groups on the substrate; iii) Chemical cross‐linking with sulfonic groups.

Pyrrole monomer was polymerized on SP via APS initiation, and Ppy‐coated SP surface was constructed. The preparation process is illustrated in **Figure** **1**a. Figure 1b shows the surface morphologies, elemental composition, and macroscopic photographs of the SP, SPPL, SPPM, and SPPH samples, while Figure 1c shows the elemental content of each group. The 3D porous skeleton of SP was wrapped with Ppy nanoparticles that are ≈100 nm in size. EDS spectra and elemental content analysis revealed the presence of nitrogen on the surface of the Ppy‐modified samples, and the nitrogen ratio gradually increased on the surfaces of SPPL, SPPM, and SPPH. The zeta potentials of SP, SPPL, SPPM, and SPPH also gradually shifted to positive values at pH = 7.4 (Figure S1, Supporting Information). These results highlight the gradient content of Ppy in SPPL, SPPM, and SPPH. Based on the macroscopic photographs of each group (the inset in the upper right corner of the EDS spectrum), the surface color changed from brown to black with increasing Ppy content.

The successful loading of Ppy onto the SP surface was verified by Fourier transform infrared (FTIR) and Raman spectroscopy, as shown in Figure 1d,e. Based on Figure 1d, weak plane deformation peaks of N—H bonds in the pyrrole rings were observed at 1041 cm^−1^ on the surface of the SPPM and SPPH samples.^[^ ^43^ ^]^ The Raman spectrum also revealed four obvious Raman peaks of Ppy on the SPPM sample surface: 1584.76 cm^−1^, 1338.32 cm^−1^, 1049.52 cm^−1^, and 987.26 cm^−1^, which corresponded to *V*_C=C_, *V*_C—C_, *δ*_C—H_, and *δ*_ring_ vibration modes, respectively (Figure 1e).^[^ ^44^ ^]^ Molecular bond simulation was used to calculate the bond energies between the SP molecular chain and pyrrole monomer, and the results are shown in Figure 1f. Pyrrole tended to form the S—O—C chemical link with the SP molecular chain due to the lowest bond energy, −105.4 kcal mol^−1^, compared to −8.05 kcal mol^−1^ of NH···O=C and −10.47 kcal mol^−1^ of NH···O—SO_3_H.

### 2.2 Physicochemical Properties of Ppy‐Coated SP

**Figure 2:**
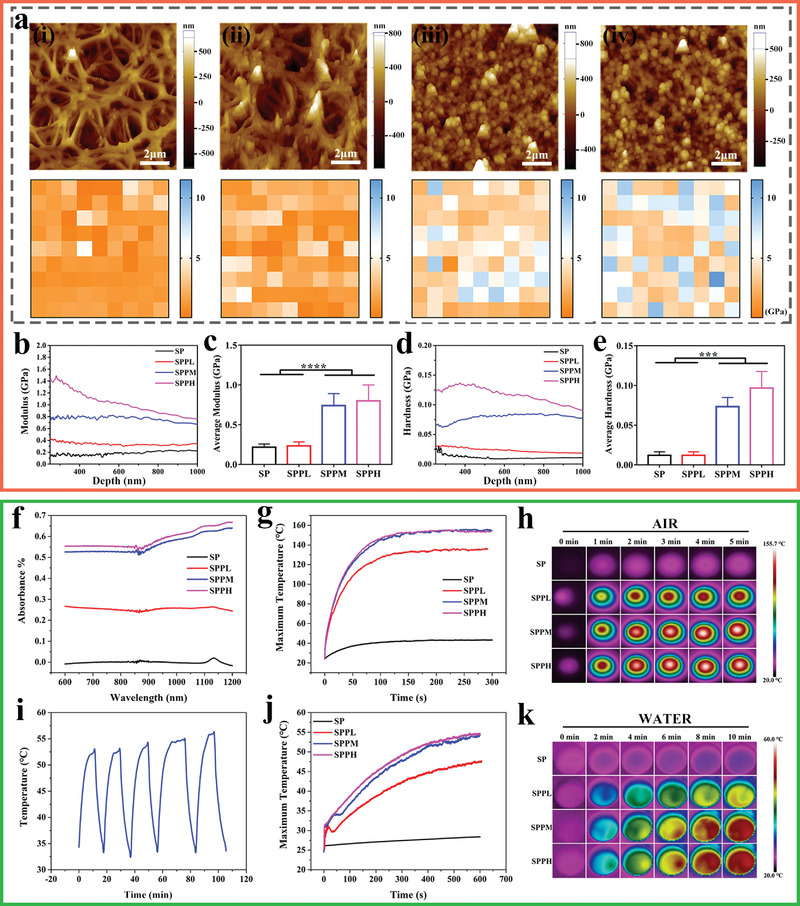
Surface physicochemical properties of each group. a) Atomic force microscope images and corresponding heat maps of elastic modulus distribution on i) SP, ii) SPPL, iii) SPPM, and iv) SPPH. b) Elastic modulus curves and c) average modulus; d) Hardness curves and e) average hardness detected by nano‐indentation tests for different samples. f) UV–vis absorption spectrum. g) Temperature change and h) Infrared thermal images of different samples under 808 nm NIR at 0.5 W cm^−2^ in air. i) Temperature variations of SPPM samples for 5 on/off cycles in 1 mL ultrapure water. j) Temperature change and k) Infrared thermal images of different samples under 808 nm NIR at 0.5 W cm^−2^ in 1 mL ultrapure water. Data represent means ± SD (*n* = 6). Statistical significance was calculated by one‐way ANOVA analysis. *p < 0.05; **p < 0.01; ***p < 0.001; ****p < 0.0001.

The surface elastic modulus distributions of different samples are shown in **Figure** **2**a. The modulus enhanced with increasing Ppy content, and the elastic moduli of the SPPM and SPPH samples were approximately fourfold higher than those of the SP and SPPL samples. Figure 2b–e reveals the modulus and hardness variation versus the depth acquired from different surfaces. The modulus and hardness values of the SPPM and SPPH samples were significantly higher than those of the SP and SPPL samples. The average modulus values for the SP, SPPL, SPPM, and SPPH samples were 0.22 ± 0.04, 0.23 ± 0.05, 0.74 ± 0.15, and 0.80 ± 0.20 GPa, respectively. The average hardness values of the SP, SPPL, SPPM, and SPPH samples were 0.012 ± 0.004, 0.012 ± 0.004, 0.073 ± 0.012, and 0.097 ± 0.021 GPa, respectively. Further, the skeleton structure of the SPPM and SPPL samples could resist ultrasound damage and be retained whereas that of the SP samples collapsed (Figure S2, Supporting Information). The bonding between the coating and substrate in the Ppy‐coated SP group was significantly stronger than that in the Ppy‐coated PEEK (Figure S3, Supporting Information). These findings verify that Ppy can effectively increase the toughness of the SP skeleton.

Figure 2f shows the UV–vis absorption spectra of different samples. Ppy‐modified samples had higher absorption than SP at wavelengths of 600–1200 nm, and the absorbance was enhanced as the Ppy content on the surface of SP was increased. The photothermal effect of various samples under 808 nm NIR irradiation was investigated. First, the temperature changes and infrared thermal images of each group were examined under NIR irradiation at a power density of 0.5 W cm^−2^ in air for 5 min (Figure 2g,h). The temperature in each group could increase from 24.8 °C within 100 s. Further, the maximum temperature of the SP, SPPL, SPPM, and SPPH samples was 40.9, 127.3, 146.1, and 147.0 °C, respectively. The temperature changes and infrared thermal images of each group were tested under NIR at the same power density in 1 mL of ultrapure water for 10 min (Figure 2j,k). The temperature in each group increased from 24.0 °C within 10 min. The maximum temperature of the SP, SPPL, SPPM, and SPPH samples was 28.5, 47.5, 54.4, and 54.7 °C, respectively. Compared with SPPH, SPPL and SPPM had lower photothermal conversion capacities. Therefore, their surface temperature perturbation occurred at lower temperatures and their temperature drops more than SPPH owing to thermal diffusion. Finally, the photothermal cycle curve of the SPPM samples was tested. Based on the results in Figure 2i, the Ppy modified samples exhibited good photothermal cycling performance.

### 2.3 In Vitro Evaluation

#### 2.3.1 Antibacterial Properties

**Figure 3:**
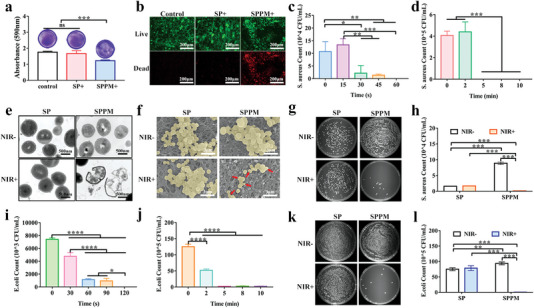
Antibacterial activity of the samples. a) Crystal violet staining and b) Live/dead staining for *S. aureus* cultured on SP and SPPM samples irradiated with NIR at a power density of 0.5 W cm^−2^ for 5 min. Bacterial colony count of *S. aureus* cultured on SPPM samples under NIR irradiation at a power density of 0.5 W cm^−2^ for different irradiation times c) in air and d) in physiological saline. e) Transmission electron microscopy images; f) Scanning electron microscope images; g) Bacterial colony plate images without dilution and h) corresponding colony count statistics of *S. aureus* cultured on SP and SPPM samples irradiated with NIR at a power density of 0.5 W cm^−2^ for 5 min. Bacterial colony statistics of *E. coli* cultured on SPPM samples after NIR irradiation at a power density of 0.5 W cm^−2^ for different times i) in air and j) in physiological saline. k) Bacterial colony plate images at 100 times dilution and l) colony count statistics of *E. coli* cultured on SP and SPPM samples irradiated with NIR at a power density of 0.5 W cm^−2^ for 5 min. Data represent means ± SD (*n* = 3). Statistical significance was calculated by one‐way ANOVA analysis. *p < 0.05; **p < 0.01; ***p < 0.001; ****p < 0.0001.

The antibacterial abilities of different samples irradiated by NIR at 0.5 W cm^−2^ are illustrated in **Figure** **3**. Bacterial biofilms stained with crystal violet were quantitatively analyzed (Figure 3a). Compared with the control and SP groups with intact biofilms, the biofilms in the SPPM group were destroyed, and the bacteria were dispersed in the well plate. Live/dead bacteria were calibrated in the control, SP, and SPPM groups (Figure 3b). The biofilms in the control and SP groups were not damaged, and only a few dead bacteria existed. In contrast, the bacterial biofilms were divided into pieces, and the bacteria in the well plate were mainly dead in the SPPM group. The rapid sterilization effects of the samples were also examined. Figure 3c and Figure S4, Supporting Information show the effect of the SPPM samples on *S. aureus* under NIR irradiation in air at different times. The number of colonies did not change significantly after irradiation with NIR radiation for 15 s compared with that without NIR stimulation. After irradiation for 30 s, the bacteriostasis rate in the SPPM group was 94%. Figure 3d and Figure S5, Supporting Information show the effect of the SPPM samples on *S. aureus* under NIR irradiation in physiological saline at different times. The antibacterial rate of the SPPM group was 90% with NIR stimulation for 5 min in physiological saline. Figure 3e,f shows the morphologies of *S. aureus* cultured on the SP and SPPM surfaces after NIR treatment. Based on the transmission electron microscope (TEM) images, the bacterial membrane was intact in the SP and SPPM groups without NIR treatment and the SP group with NIR treatment, while the bacterial membrane in the SPPM group with NIR treatment was broken and their internal matrix escaped via leaking (Figure 3e). The SEM images show similar results to the TEM results (Figure 3f). After NIR treatment, the SPPM sample surface had fewer bacteria, and their morphologies were concave from the center to the interior. Figure 3g,h shows the bacterial colony plate images and corresponding statistical results of the SP and SPPM groups under NIR irradiation for 5 min, while Figure S6, Supporting Information contains images of the SPPL and SPPH groups. Ppy‐modified samples could reduce bacteria cultured on the surface after NIR treatment compared to those without NIR treatment. Both SPPM and SPPH samples irradiated with NIR displayed good antibacterial abilities against *S. aureus*, whereas the SPPL group with NIR treatment had no significant effect compared with SP group.

Figure 3i–l shows the effect of the SP and SPPM samples with NIR simulation against *E. coli* at a power density of 0.5 W cm^−2^ at different times. The antibacterial rate of the SPPM group was more than 87% after NIR treatment for 60 s in air (Figure 3i and Figure S7, Supporting Information). The antibacterial rate of the SPPM group was greater than 90% after NIR stimulation for 5 min in physiological saline (Figure 3j and Figure S8, Supporting Information). Figure 3k,l shows the bacterial colony plate images and corresponding counting results of the SP and SPPM groups with NIR for 5 min, and the images of the SPPL and SPPH groups are shown in Figure S9, Supporting Information. Ppy‐modified samples displayed excellent antibacterial abilities against *E. coli* after NIR irradiation compared to the SP samples. Further, there was no significant difference between the SP group with and without NIR irradiation. The antibacterial rate in the SPPM and SPPH groups irradiated with NIR was greater than 90%, whereas in the SPPL group irradiated with NIR, this rate was only ≈60%.

#### 2.3.2 Inflammatory Response and Phagocytosis Efficiency of Macrophages

**Figure 4:**
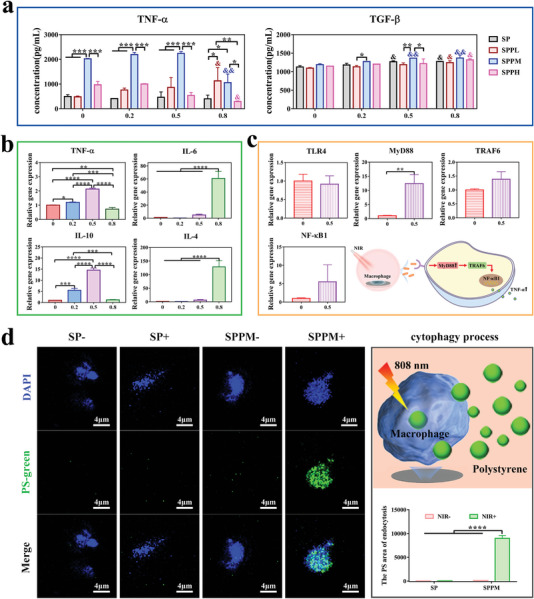
Inflammatory responses and phagocytosis efficiency of macrophages cultured on samples with NIR irradiation. a) Inflammatory cytokines release levels from macrophages cultured on each group under NIR treatment at different power densities (Unit: W cm^−2^). &: Compare with the same group without NIR treatment; *: Different samples compare with each other at the same power density. b) Expression of inflammation‐related genes in macrophages cultured on the SPPM samples under NIR treatment at different power densities (Unit: W cm^−2^). c) Related genes expression in TLR4–MyD88–NF‐*κ*B1 signaling pathway of the SPPM group under NIR treatment (Unit: W cm^−2^). d) The phagocytosis rate of macrophages cultured on SP and SPPM samples for PS microspheres with or without NIR treatment at 0.5 W cm^−2^ and the schematic diagram of cytophagocytosis process. Data represent means ± SD (*n* = 3). Statistical significance was calculated by *t*‐test, one‐way ANOVA analysis, two‐way ANOVA analysis, and Tukey's multiple comparison tests. *p < 0.05; **p < 0.01; ***p < 0.001. ^&^ *p* < 0.05; ^&&^ *p* < 0.01.

The proliferation of macrophages cultured on different samples was evaluated. Based on the results, none of the samples were cytotoxic (Figure S10, Supporting Information). The cells cultured on Ppy‐modified samples had many pseudopodia, whereas those cultured on SP samples had a spherical shape (Figure S11, Supporting Information). **Figure** **4**a shows the secretion of inflammatory cytokines by the macrophages in each group under NIR stimulation at different power densities. TNF‐*α* and TGF‐*β* secretion in the SPPM group was the highest among the four groups after NIR stimulation at different power densities. The highest TNF‐*α* and TGF‐*β* secretion was observed in the SPPM group stimulated with NIR at 0.5 W cm^−2^ among four power densities. However, the SPPL and SPPH groups showed no significant difference compared with the SP group stimulated with NIR at 0, 0.2, 0.5 W cm^−2^ for TNF‐*α* and TGF‐*β* secretion. Under 0.8 W cm^−2^ NIR treatment, the SPPL group promoted TNF‐*α* secretion and the SPPH group had no effect compared to the SP group. In addition, the regulation of cytokine secretion by NIR irradiation was not significant in the SPPL and SPPH groups. Only the NIR treatment at 0.8 W cm^−2^ slightly promoted TNF‐*α* and TGF‐*β* secretion compared with that at 0 W cm^−2^ in the SPPL group, while the NIR treatment at 0.8 W cm^−2^ decreased TNF‐*α* secretion and enhanced TGF‐*β* secretion compared with that at 0 W cm^−2^ in the SPPH group. Figure S12, Supporting Information shows the expression of inflammation‐related genes in macrophages cultured on different samples without NIR irradiation. The SPPM group showed significant inhibition of TNF‐*α* and IL‐6 gene expression compared with the SPPL and SPPH groups without NIR stimulation. There was no significant difference in the regulation of TNF‐*α* and IL‐6 gene expression between the SPPM and SP groups. Furthermore, the SPPM group showed significantly upregulated expression of the IL‐10 gene compared to that in the other three groups. To determine the regulation of inflammatory gene expression in macrophages cultured on samples under NIR stimulation at different power densities, SPPM samples were selected based on their superior anti‐inflammatory effects; the results are shown in Figure 4b. The expression levels of the TNF‐*α* and IL‐10 genes were upregulated with increasing power density of NIR treatment up to 0.5 W cm^−2^. Further, TNF‐*α* gene expression was downregulated under 0.8 W cm^−2^ NIR treatment compared with that obtained without NIR irradiation. Additionally, the expression levels of the IL‐6 and IL‐4 genes were significantly upregulated under 0.8 W cm^−2^ NIR treatment compared with that in the other three groups with no significant difference. To investigate the mechanism that SPPM group enhanced TNF‐*α* secretion under 0.5 W cm^−2^ NIR treatment, the related gene expression levels in TLR4–MyD88–NF‐*κ*B1 signaling pathway were detected; and the results are shown in Figure 4c. There was no significant difference between both groups for TLR4 receptor expression, while MyD88, TRAF6, and NF‐*κ*B1 genes expression was substantially enhanced in the SPPM group under 0.5 W cm^−2^ NIR stimulation compared with that in the SPPM group without NIR treatment.

The phagocytic efficiency of macrophages cultured on SP and SPPM samples was investigated; the results are provided in Figure 4d. Macrophages cultured on SP samples had low phagocytosis efficiency, regardless of NIR stimulation. The phagocytosis efficiency in the SPPM group stimulated with NIR was significantly increased, whereas that in the SPPM group without NIR was not significantly different from that in the SP group. Overall, SPPM could regulate on‐off phagocytosis of macrophages by controlling NIR. Additionally, stimulation with 0.5 and 0.8 W cm^−2^ NIR induced an increase in the M1 proportion, whereas stimulation with 0 and 0.2 W cm^−2^ led to a higher M2 proportion in the SPPM group. However, NIR had no effect on the proportion of M1 macrophages in the SP group (Figure S13, Supporting Information). The SPPM samples possessed superior antibacterial properties, on‐off phagocytosis, and switchable macrophage activation. Therefore, SPPM samples were selected to determine the in vivo effects.

### 2.4 In Vivo Evaluation

#### 2.4.1 Antibacterial Properties

**Figure 5:**
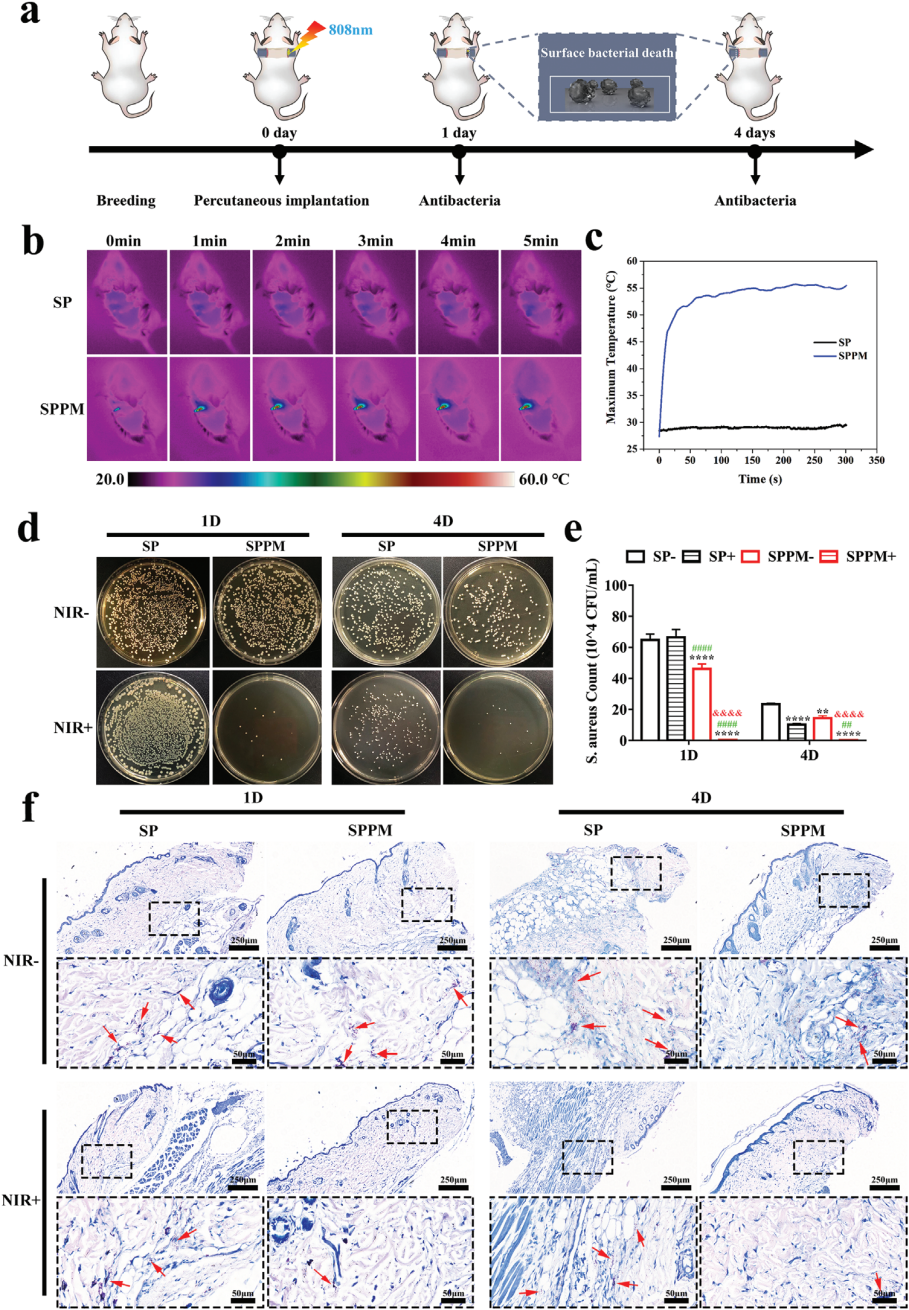
In vivo Antibacterial. a) Schematic diagram of photothermal antibacterial process of materials. b) Infrared thermal images and c) Temperature changes of implantation site in mice under NIR irradiation at a power density of 0.5 W cm^−2^. d) Bacterial colony plate images at ten times dilution and e) colony count statistics of *S. aureus* in each group after 1 and 4 days of implantation. f) Giemsa staining of surrounding tissues 1 and 4 days after implantation in different groups. Deep purple: bacterial colonies. Data represent means ± SD (*n* = 3). Statistical significance was calculated by two‐way ANOVA analysis and Tukey's multiple comparison tests. *p < 0.05; **p < 0.01; ***p < 0.001; ****p < 0.0001 compared with SP‐. ^&^ *p* < 0.05; ^&&^ *p* < 0.01; ^&&&^ *p* < 0.001; ^&&&&^ *p* < 0.0001 compared with SPPM‐. ^#^ *p* < 0.05; ^##^ *p* < 0.01; ^###^ *p* < 0.001; ^####^ *p* < 0.0001 compared with SP+.

To investigate the photothermal antibacterial properties of different samples in vivo, a percutaneous implantation infected model was constructed on the backs of mice; the schematic diagram is shown in **Figure** **5**a. Figure 5b,c shows the infrared thermal images and temperature changes of the SP and SPPM samples in mice under NIR irradiation at a power density of 0.5 W cm^−2^. The SP group was maintained at ≈28 °C. Of note, the temperature of the SPPM group increased to ≈55 °C within 50 s. Figure 5d,e shows the bacterial colony plate images and the corresponding colony count of different groups after implantation for 1 and 4 days. After implantation for 1 day, the number of bacteria cultured on SPPM samples was slightly less than that cultured on SP samples without NIR treatment. NIR irradiation helped to further reduce the number of bacteria cultured on the SPPM samples, whereas there was no significant effect on the SP group. The antibacterial rate in the SPPM group with NIR irradiation was greater than 95%. After 4 days of implantation, the SPPM group with NIR treatment maintained a good bacteriostasis rate, which was better than that of the other three groups. Giemsa staining was used to visualize the bacteria in the surrounding tissues of each group after implantation; the results are shown in Figure 5f. The bacterial colonies were stained with dark purple granules. The SPPM group with NIR reduced the number of bacteria in the surrounding tissues over time.

#### 2.4.2 Phagocytic Signaling Pathways

**Figure 6:**
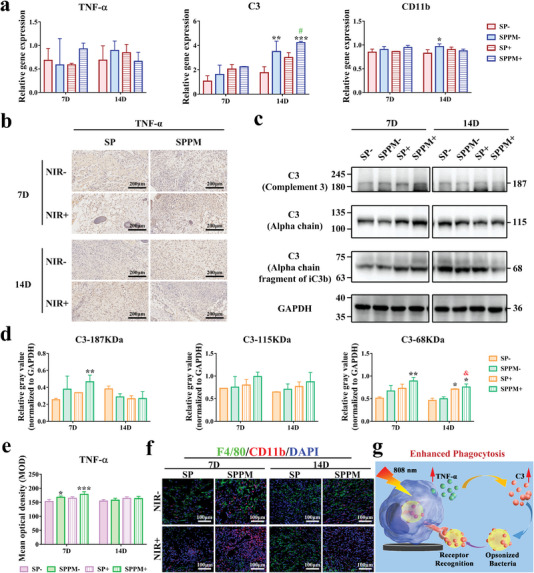
Phagocytic signaling pathways. a) Gene expression in phagocytic signaling pathways. “‐”: the material without NIR irradiation; “+”: the material under NIR irradiation at a power density of 0.5 W cm^−2^. b) Immunohistochemical staining for TNF‐*α* protein. c) Western blot characterization of C3 complement protein and branched chain and d) quantitative analysis of corresponding protein expression. e) Quantitative analysis of TNF‐*α* protein by immunohistochemical staining. f) Macrophage, CD11b protein, and nuclear markers. (F4/80: green; CD11b: red; Nucleus: blue) in the surrounding tissues after 7 and 14 days of implantation in the percutaneous implantation infected model. g) Schematic diagram of the mechanisms of phagocytosis pathway. Data represent means ± SD (*n* = 3). Statistical significance was calculated by two‐way ANOVA analysis and Tukey's multiple comparison tests. *p < 0.05; **p < 0.01; ***p < 0.001 compared with SP‐. ^&^ *p* < 0.05 compared with SPPM‐. ^#^ *p* < 0.05 compared with SP+.

Gene and protein expression in phagocytic‐related pathways were characterized in the infected group; the results are shown in **Figure** **6**. Figure 6a shows the gene expression in the phagocytosis pathway. TNF‐*α* gene expression in the SPPM group was upregulated by NIR irradiation and was higher than that in the other three groups after day 7. After day 14, the NIR stimulation effect on the SPPM group disappeared, and the trend of TNF‐*α* gene expression was SPPM+ ≈SP‐ < SP+ ≈ SPPM‐. C3 gene expression in the SPPM group was higher than that in the SP group, and NIR stimulation slightly increased its expression in both groups on days 7 and 14. CD11b gene expression was also upregulated in the SPPM group compared with that in the SP group on day 7. As the time extended to 14 days, CD11b gene expression in the SPPM group was downregulated compared to that in the SP group with NIR irradiation. Figure 6b,e shows the qualitative and quantitative analyses of the TNF‐*α* protein, respectively. TNF‐*α* expression in the SPPM group was significantly higher than that in the SP group, and its expression slightly increased through NIR stimulation on day 7. The positive area (brown) for TNF‐*α* in the SPPM group with NIR irradiation was lower than that in the other groups after day 14. Figure 6c,d shows the expression levels of the C3 protein. The SPPM group had high expression of C3, its *α* chain, and the *α* chain fragment of iC3b compared to that in the SP group, and NIR stimulation slightly increased C3 expression at day 7. After day 14, the effect of NIR irradiation on C3 expression was abolished. The trends for the *α*‐chain of C3 and *α*‐chain fragment of iC3b expression on day 14 were similar to those on day 7. Figure 6f shows the labeling of macrophages (F4/80, green), CD11b proteins (red), and nuclei (blue). The number of macrophages in the group with NIR irradiation was less than that in the group without NIR stimulation, and the SPPM group had fewer macrophages than the SP group with NIR stimulation at day 7. After day 14, the number of macrophages in the SPPM group with NIR irradiation was reduced, whereas that in the other three groups was increased. CD11b expression in the SPPM group was higher than that in the SP group, and NIR stimulation increased its expression in the SPPM groups on day 7. CD11b expression in the SPPM group with NIR irradiation was reduced and lowered than that in the other groups after day 14. However, NIR stimulation had no significant effect on the SP group. Figure 6g shows the mechanism of action of the phagocytic pathway. When the samples were stimulated with NIR, macrophages cultured on the samples secreted excessive amounts of TNF‐*α*. TNF‐*α* subsequently increased C3 and CD11b expression. Finally, the highly expressed C3 could effectively opsonize bacteria to bind to the CD11b receptor on the surface of macrophages, thereby promoting phagocytosis.

#### 2.4.3 Tissue Healing Evaluation in a Percutaneous Implantation Infected Model

**Figure 7:**
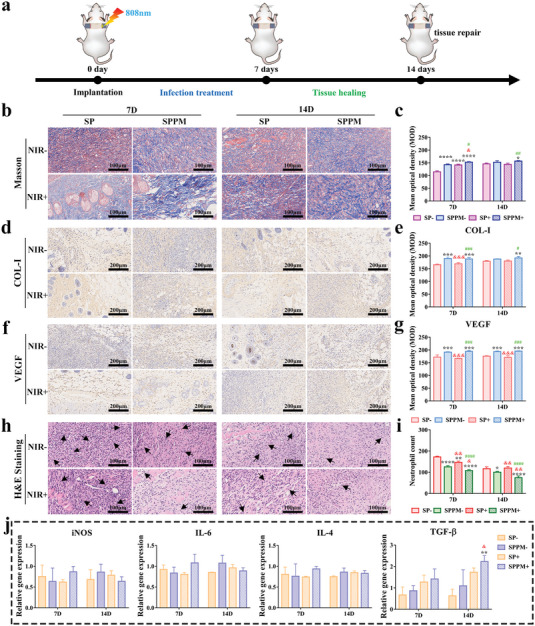
Tissue healing evaluation in the percutaneous implantation infected model. a) Schematic diagram of tissue healing observation using percutaneous implantation infected model. NIR at a power density of 0.5 W cm^−2^ and stimulation for 5 min. b) Masson staining image and c) corresponding quantitative analysis. d) Immunohistochemical staining images of COL‐I and e) corresponding quantitative analysis. f) Immunohistochemical staining images of VEGF and g) corresponding quantitative analysis. h) H&E staining images and i) neutrophil count. j) Inflammation‐related genes expression of surrounding tissues in each group after implantation of 7 and 14 days. Data represent means ± SD (*n* = 3). Statistical significance was calculated by two‐way ANOVA analysis and Tukey's multiple comparison tests. *p < 0.05; **p < 0.01; ***p < 0.001; ****p < 0.0001 compared with SP‐. ^&^ *p* < 0.05; ^&&^ *p* < 0.01; ^&&&^ *p* < 0.001; ^&&&&^ *p* < 0.0001 compared with SPPM‐. ^#^ *p* < 0.05; ^##^ *p* < 0.01; ^###^ *p* < 0.001; ^####^ *p* < 0.0001 compared with SP+.

Tissue healing was observed after percutaneous implantation for 7 and 14 days; a schematic diagram of the treatment process is shown in **Figure** **7**a. The distribution of collagen fibers in tissues was first observed and quantified using Masson staining (Figure 7b,c). The SPPM group had a higher collagen fiber content than the SP group on days 7 and 14, and NIR stimulation increased collagen fiber distribution in the SPPM and SP groups on day 7. The collagen fiber content in the SPPM group was still higher than that in the SP group under NIR treatment. COL‐I and VEGF are important indicators of collagen secretion and vascular formation in the tissues. Figure 7d‐g provides the results of qualitative and quantitative analyses of COL‐I and VEGF in the tissues around the implants. The SPPM group had higher COL‐I and VEGF expression levels than the SP group with or without NIR treatment after implantation for 7 and 14 days. NIR stimulation increased the positive area (brown) of COL‐I and VEGF in the SPPM group on day 14 but had no effect in the SP group. Figure 7h–j shows the inflammation in the tissue surrounding the implants. The tissues were stained with H&E, and the inflammatory cells were statistically analyzed (Figure 7h,i). The number of neutrophils in the SPPM group was lower than that in the SP group, and NIR stimulation reduced the number of cells in each group after day 7. As the time extended to 14 days, the number of inflammatory cells in all groups decreased, and the trend among all groups was the same as that at day 7. The expression of inflammation‐related genes was also analyzed, and the results are shown in Figure 7j. The SPPM group with NIR stimulation showed significantly upregulated TGF‐*β* expression compared to that of the other groups on day 14. Furthermore, the effect of NIR stimulation on the macrophage phenotype was evaluated, and the results are shown in Figure S14, Supporting Information. The M1/M2 ratio in the SPPM group was higher than that in the SP group, and NIR treatment increased the M1/M2 ratio in each group after day 7. After day 14, M1/M2 in each group decreased, and the SPPM group with NIR treatment had the lowest M1/M2 rate relative to that of the other groups.

#### 2.4.4 Tissue Healing Evaluation in a Percutaneous Implantation Model

**Figure 8:**
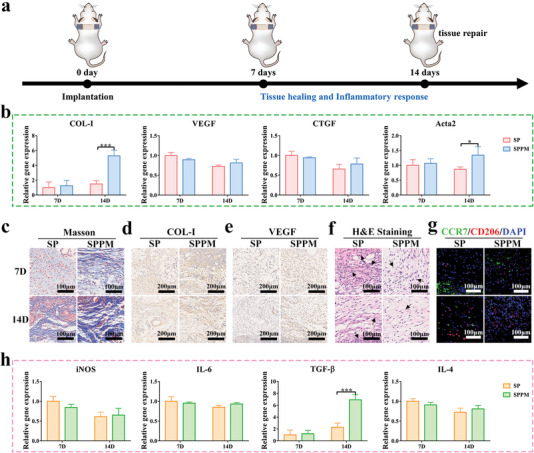
Tissue healing in the percutaneous implantation model. a) Schematic diagram of model construction process. b) Tissue healing related gene expression levels after implantation for 7 and 14 days. c) Masson staining images; Immunohistochemical staining images of d) COL‐I and e) VEGF; f) H&E staining images; g) Immunofluorescence staining images of labeled macrophage phenotype (M1: CCR7: Green; M2: CD206: Red; DAPI: Blue) in surrounding tissues of SP and SPPM groups at 7 and 14 days. h) Expression levels of inflammation‐related genes at 7 and 14 days. Data represent means ± SD (*n* = 3). Statistical significance was calculated by two‐way ANOVA analysis and Tukey's multiple comparison tests. *p < 0.05; **p < 0.01; ***p < 0.001.

Tissue healing was also investigated in a percutaneous implantation model in this study. As shown in **Figure** **8**a, the materials were implanted into the backs of mice, which were administered food for 7 and 14 days to observe tissue healing and inflammatory response at the late stage. All implants displayed good biosafety (Figure S15, Supporting Information). Figure 8b shows the expression levels of the tissue‐healing‐related genes. The expression levels of the COL‐I and Acta2 genes in the SPPM group were significantly higher than those in the SP group after implantation for 14 days. The in vitro experiments also revealed that SPPM samples had good biocompatibility, promotion of spread, and COL‐I, TGF‐*β*1, and CTGF gene expression in L929 (Figures S16–S19, Supporting Information). Figure 8c shows the distribution of collagen and muscle fibers, marked by Masson staining. The distribution density of collagen fibers in the SPPM group was higher on days 7 and 14. In addition, the collagen fiber density in the SP and SPPM groups increased significantly with the extension of the implantation time. Figure 8d,e shows the COL‐I and VEGF protein‐positive areas (brown areas), respectively. The expression of these two proteins in the SPPM group was higher than that in the SP group on days 7 and 14. The inflammatory response during the healing stage was then observed. The tissues were stained with H&E and the inflammatory cells were statistically analyzed (Figure 8f). Neutrophils in the SPPM group were lower than those in the SP group on days 7 and 14. The number of inflammatory cells in each group gradually subsided with increasing implantation time. Figure 8g shows the pro‐inflammatory M1 (CCR7, green) and anti‐inflammatory M2 (CD206, red) markers in tissues. After implantation for 7 days, the M2/M1 ratio in the SPPM group was higher than that in the SP group. At day 14, M2/M1 in all groups increased, and the M2/M1 trend remained the same between the SPPM and SP groups. Figure 8h shows the expression of the genes associated with inflammation. The SPPM group showed significantly upregulated anti‐inflammatory TGF‐*β* gene expression at day 14 compared to the SP group.

## 3 Discussion

In this study, a Ppy coating was constructed on SP through a highly efficient confinement reaction in situ on the porous framework of the SP; this process was inspired by the formation of a structure in which steel bars are wrapped with concrete. In particular, under the action of ammonium persulfate (APS), pyrrole monomers are oxidized and lose electrons to become cationic radicals, which are introduced into the 3D porous framework and form stable chemical bonds with the electron‐withdrawing sulfonic groups on the SP surface. Ppy long‐chain molecules are efficiently formed on the porous framework via a disproportionation reaction in a limited porous domain.^[^ ^45^ ^]^ The protonation reaction of the pyrrole monomer occurs in a weakly acidic environment owing to the residual acidic solution after sulfonation treatment, which facilitates the rapid and uniform nucleation of Ppy on the framework containing sulfonic groups through electrostatic adsorption and hydrogen bonding. The pyrrole monomer subsequently grew and covered the 3D porous skeleton. Ppy simulates cement hydration and forms nanoparticles wrapped on the soft skeleton of SP to become the overall structure; this mimics the reinforced concrete structure used in house construction, resulting in enhanced mechanical properties. Among them, polymerized Ppy provides strong compressive strength for the surface, while the polymer PEEK ensures a certain tensile strength for the whole. The molecular bond simulation results indicated that the S‐O‐C chemical link is the preferable bonding form between the chain end of Ppy and the sulfonate group of SP. Compared to the other two forms, NH···O=C and NH···O—SO_3_H, the chemical bond was stronger than the hydrogen bond. Hence, Ppy showed superior adhesion to SP (Figure S3, Supporting Information). The strong chemical bond also helped with the formation of a tight encapsulation of the porous skeleton on SP, which ultimately enhances the surface modulus and hardness of SP (Figure 2a–e and Figure S2, Supporting Information).

Ppy has excellent photostability and photothermal conversion efficiency and can achieve good thermal conductivity through electrons owing to its large conjugated structure.^[^ ^46^, ^47^ ^]^ Ppy‐coated SP has excellent photothermal effects in air (140 °C after 70 s) and water (54.4 °C after 10 min) under NIR irradiation at a power density of 0.5 W cm^−2^, and can quickly reach maximum temperature platforms (Figure 2); this is closely related to the synergistic effect between the excellent photothermal effect of Ppy and heat preservation of PEEK. On one hand, the photothermal conversion efficiency of Ppy‐coated SP was superior to that of the PDA@Ppy nanoparticle‐coated epoxy resin and gold nanorod‐modified hydrogels under 808 nm NIR treatment. In particular, the epoxy resin coating with PDA@Ppy reached the maximum temperature of 110 °C for 7 min in air and 53 °C for 10 min in water after NIR irradiation at a power density of 2.0 W cm^−2^. The hydrogels containing gold nanorods can increase to 48 °C after NIR irradiation at a power density of 0.5 W cm^−2^ for 10 min.^[^ ^48^, ^49^ ^]^ On the other hand, PEEK has a lower thermal conductivity (0.28 W mK^−1^) than metallic medical materials, such as titanium (15.24 W mK^−1^), which can induce converted heat to concentrate on the surface of the porous skeleton, as PEEK is used as an interior support material, thereby rapidly increasing the surface temperature.

Studies have shown that shortening the irradiation time or lowering the NIR power can avoid thermal damage caused by PTT.^[^ ^50^, ^51^, ^52^ ^]^ In this study, the Ppy‐modified surface exhibited a heat aggregation effect and could serve as an effective means of rapid sterilization. Ppy‐coated SP can lead to bacterial content leakage, achieve over 90% bacteriostatic rate against *S. aureus* and destroy the bacterial biofilms on the implants surface in a shorter time (30 s in air and 5 min in water) at a lower power density (0.5 W cm^−2^) of NIR (Figures 3 and 5, and Figures S4–S9, Supporting Information). Hybrid elastomers have been developed with rapid photothermal sterilization at a power density of 1.0 W cm^−2^ for 60 s in air and 2.0 W cm^−2^ for 10 min in aqueous solutions.^[^ ^53^ ^]^ Red phosphorus‐coated titanium can kill bacteria at a power density of 1.0 W cm^−2^ for 10 min.^[^ ^54^ ^]^ Ppy‐coated SP has a superior rapid photothermal sterilization compared to recent hybrid elastomers and modified titanium.

Dissociative bacteria are difficult to kill in vivo; therefore, intelligent on‐off phagocytosis by macrophages is essential for the removal of residual bacteria and superior tissue healing.^[^ ^26^, ^28^ ^]^ In this study, Ppy‐coated SP could enhance the phagocytic activity and M1 polarization of macrophages by activating C3 and its receptor, CD11b, with NIR (optimal 0.5 W cm^−2^) irradiation, which facilitates the removal of free bacteria. Low PTT stimulation activates local tissue damage‐associated molecular patterns (DAMPs), which activate the complement and receptor system through classical and lectin pathways and induce pro‐inflammatory M1 through TLR4–MyD88–NF‐*κ*B1 signaling pathway to secrete abundant TNF‐*α*, IL‐1*β*, and other factors.^[^ ^55^, ^56^, ^57^, ^58^, ^59^, ^60^ ^]^ TNF‐*α* stimulates the expression of C3 and its receptor, which can effectively opsonize invading bacteria, thereby driving the connection between bacteria and the phagocytic cell surface receptor, CD11b, to promote phagocytosis and respiratory bursts. In turn, the binding of C3 to its receptor promotes M1 switching.^[^ ^26^, ^28^, ^61^ ^]^ Activated M1 releases pro‐inflammatory factors, such as interferon‐*γ* (IFN‐*γ*), and reactive oxygen intermediates (ROI), such as nitric oxide (NO) and metalloproteinase 12 (MMP12), to enhance bacterial damage.^[^ ^62^, ^63^ ^]^ However, the phagocytic activity and M1 polarization of macrophages decreased with the closure of NIR after bacterial clearance (Figure 4; Figures S13 and S14, Supporting Information). Furthermore, previous work revealed that PTT at a mild temperature (41 ± 1 °C) can further accelerate the transition of the macrophages from the pro‐inflammatory (M1) phenotype to the anti‐inflammatory (M2) phenotype. The phagocytosis of macrophages can be turned off as NIR is turned on with more subtle temperature control and will be assessed in the future.^[^ ^64^ ^]^

After bacterial removal, a switch from M1 to M2 macrophage marks the transition from inflammation to tissue regeneration.^[^ ^65^, ^66^ ^]^ M2 macrophages produce highly expressed anti‐inflammatory cytokines, resistance tin‐like molecules as repair mediators, and tissue growth factors to promote vascular maturation and maintain vascular and tissue proliferation in the proliferation stage of tissue cells, such as fibroblasts.^[^ ^36^ ^]^ Ppy‐coated SP can reduce the number of inflammatory cells in the surrounding tissues, increase the M2 proportion, and significantly upregulate the expression of the pro‐healing TGF‐*β* gene; this may be because the surface properties of Ppy‐coated SP have a regulatory effect on cells. Cell morphology and response can be determined by mechanosensing, mechanotransduction, and mechanoresponses.^[^ ^67^ ^]^ In particular, the surface with higher elastic modulus can promote macrophage binding, phagocytosis, and M2 polarization.^[^ ^68^, ^69^ ^]^ Hence, SPPM with a higher surface modulus tends to induce M2 with anti‐inflammatory properties compared with SP and SPPL with a lower modulus. However, SPPH with the highest modulus among the four groups causes a pro‐inflammatory response compared with SPPM as the surface with a 3D porous structure reduces inflammation more easily than a flat surface (Figure 4).^[^ ^70^ ^]^ The surface properties had a similar effect on L929 (Figure S19, Supporting Information). M2 induced by Ppy‐coated SP ultimately cooperates with L929 to promote tissue reconstruction at a later stage in the percutaneous implantation model and percutaneous implantation infected model. Previous work revealed that the presentation of ligands on material surfaces, such as RGD, IL‐4, and IL‐13, can regulate macrophage adhesion and polarization to M2. The Ppy coating of SP has superior drug loading ability owing to its 3D porous surface and high potential in NIR response to drug release, which may be a useful strategy for combining Ppy coating with bioactive agents to further precisely regulate immune responses around implants.^[^ ^71^, ^72^, ^73^ ^]^

## 4 Conclusions

In this study, Ppy grew in situ in the 3D porous limited domain of SP, which improved the surface mechanical properties of SP. At the bacterial infection stage, Ppy‐coated SP could respond to 808 nm NIR stimulation to produce surface heat accumulation, thereby killing bacteria and even rapidly destroying the bacterial biofilms. Ppy‐coated SP with NIR stimulation could also enhance phagocytosis activity and M1 activation of macrophages on the implant surface by increasing C3 and its receptor, CD11b, expression to complete the elimination of dissociative bacteria in vivo. At the bacteria‐controlled stage, phagocytosis can be shut down by turning off NIR irradiation. Moreover, macrophages switch to M2 owing to the higher surface modulus without NIR treatment, which increases COL‐I and VEGF expression and regulates fibroblasts to promote tissue healing. This study provides a candidate material for percutaneous implants through on‐off phagocytosis and switchable macrophage activation stimulated with NIR.

## 5 Experimental Section

### Sample Preparation and Characterization

Medical PEEK materials were purchased from Jiangsu Jun Hua Company and machined to disk with Φ 12.5 mm × 1 mm and cylinder with Φ 2 mm × 18 mm. The PEEK was polished and cleaned with acetone, alcohol, and ultra‐pure water for 10 min respectively.

The 3D porous structure was obtained on PEEK surface by sulfonation with concentrated sulfuric acid, as described in previous work,^[^ ^74^ ^]^ and the sample was denoted as SP. SP samples were put into the 100 mL alcohol solution (alcohol: water = 1:1 V/V) containing 0.1 m APS (Aladdin). After 10 min of mixing, 1 mL pyrrole (Aladdin) monomer was added. A sustained stir was applied to get a uniform modification of the surface. The samples treated for 3 and 5 min were denoted as SPPM (SP coated with medium Ppy content) and SPPH (SP coated with high Ppy content), respectively. In addition, in order to slow the reaction rate and obtain the coating with lower Ppy content, the SP samples and APS were put into the recycled solution that has been used to prepare SPPH samples. The remaining pyrrole monomer in such solution was lower than the initial mixed solution of pyrrole and APS for SPPH preparation. After stirring for 30 min, the modified samples were prepared successfully, and denoted as SPPL (SP coated with low Ppy content). All samples were cleaned with ultrapure water, and then treated with boiling water for 30 min to remove the residual chemical reagent.

The surface morphologies and element composition were tested by field‐emission scanning electron microscopy (Magellan 400, FEI, USA) and energy dispersive X‐ray spectroscopy (EDS; SDD550, IXRF, USA), respectively. The surface functional groups were detected by recording FTIR (Tensor 27, Bruker, Germany) spectroscopy and Raman spectra (GX‐PT‐1500 (150)). The surface elastic modulus and hardness were tested by atomic force microscope (AFM, Park XE7, Korea) and G200 nano‐indenter. Ultraviolet–visible absorption spectrum was tested by an ultraviolet–visible spectrophotometer (UV–vis, Lambda 750; Perkin Elmer, Foster City, CA, USA). And the temperature changes, infrared thermal images, and photothermal cycle curve in air and 1 mL water were tested by 808 nm laser (MDL‐H) at a density of 0.5 W cm^−2^ and infrared thermal imager (Fotric 285s, USA).

The theoretical calculations were performed via the Gaussian 16 suite of programs. The structure of the SP (*n* = 1) and its complex with pyrrole were fully optimized at the B3LYP/6‐31+G (d, p) level of theory. The vibrational frequencies of the optimized structure were carried out at the same level. The interaction energies of the optimized structures between SP (*n* = 1) and pyrrole were calculated and the basis set superposition error (BSSE) correction was also included.

### Bacterial Culture

Gram‐positive Staphylococcus aureus (*S. aureus*, ATCC 25923) and gram‐negative Escherichia coli (*E. coli*, ATCC 25922) were used to evaluate the antibacterial properties of samples. Tryptic Soy Broth medium and Luria‐Bertani medium were used to culture *S. aureus* and *E. coli*, respectively. The samples were sterilized with 75 vol% alcohol for 2 h and then dried for further studies. The bacterial inoculation density was 1 × 10^7^ CFU mL^−1^.

### Bacterial Biofilms Destruction

The bacteria were seeded in 24‐well plates and incubated at 37 °C for 24 h to form bacterial biofilms. The planktonic bacteria in the orifice plate were washed and sucked out with physiological saline. 500 µL fresh saline was added, SP and SPPM samples were placed on the top of bacterial biofilms in different wells, respectively, and the plate bottom was irradiated with NIR at 0.5 W cm^−2^ for 5 min. The samples were removed and the bottom bacteria were gently washed with physiological saline subsequently. 1 wt% crystal violet staining solution and Live/Dead BacLight kit (L13152, Thermo Fisher Scientific Inc., USA) were used for bacterial biofilms staining and bacterial live/dead calibration, respectively. After overnight dyeing with crystal violet solution, the residual dye was cleaned with ultrapure water and the biofilms were photographed after drying. Then, 500 µL 75 vol% alcohol solution was added to each well, and the absorbance at wavelength 590 nm was tested by Cytation 5 Multi‐mode Reader to evaluate the number of remaining bacteria. The live/dead bacteria in the above biofilms were stained, and the fluorescence images were observed with the fluorescence microscope (Olympus, Japan). The wells without NIR irradiation and sample treatment were denoted as controls.

### Plate Colony Counting

Bacterial plate colony counting experiment was used to evaluate the antibacterial performance of the samples. Bacteria were cultured on SP, SPPL, SPPM, and SPPH samples for 24 h. Then the surface of the sample with bacteria was irradiated with 808 nm NIR at a power density of 0.5 W cm^−2^ in saline medium for 5 min, and the groups without NIR irradiation were denoted as control. After cleaning with physiological saline, the bacteria were separated from the samples. 100 µL bacterial suspension with appropriate dilution ratio was added to standard agar culture plate and cultured for 18 h at 37 °C. Finally, the agar plates were photographed and bacterial colonies were counted.

To test the rapid sterilization effect of samples, bacteria were cultured on SPPM samples for 24 h. After that, the samples with *S. aureus* were treated with NIR at a power density of 0.5 W cm^−2^ in physiological saline for 0, 2, 5, 8, and 10 min, and in air for 0, 15, 30, 45, and 60 s, respectively. Similarly, the samples with *E. coli* were treated with NIR in physiological saline for 0, 2, 5, 8, and 10 min, and in air for 0, 30, 60, 90, and 120 s, respectively. Then the bacterial colony counting method was applied as same as above.

In addition, plate colony counting method was also used to evaluate the antibacterial performance in vivo. Specifically, the mice were sacrificed by excessive anesthesia and the cylinder samples were collected for plate colony counting after 1 and 4 days of implantation.

### Bacterial Morphology Observation

The other samples with bacteria were rinsed and then the bacteria were fixed at 4 °C with 2.5 vol% glutaraldehyde solution (1 mL) after NIR treatment in physiological saline for 5 min. Gradient ethanol solutions (30, 50, 75, 90, 95, and 100 V/V%) and ethanol‐hexamethyldisilazane mixture solution (2:1, 1:1, 1:2, and 0:1, volume ratio) were used to dehydrate the bacteria cultured on samples for 10 min each time. Finally, a scanning electron microscope (SEM, S3400, HITACHI, Japan) was used to observe the bacterial morphologies on the sample's surface.

Similarly, the bacteria were separated from SP and SPPM samples surface and fixed with 1 mL 4% paraformaldehyde after NIR treatment in physiological saline for 5 min. After centrifugation, bacteria were pre‐embedded with agarose and fixed with 1% Osmium Tetraoxide (OsO_4_, Ted Pella Inc) in phosphate buffer solution (PBS) for 2 h at room temperature. Then, they were dehydrated with 30%‐50%‐70%‐95%‐100%‐100% alcohol for 20 min each and 100% acetone for 15 min each. Afterward, resin blocks were prepared by osmosis embedding and polymerization process and cut to 60–80 nm thin. The cells were fished out onto the 150 meshes cuprum grids. Finally, the cuprum grids were observed under TEM (HT7800/HT7700, HITACHI, Japan) and images were taken.

### Cell Culture

The mouse‐derived mononuclear macrophage leukemia cells (RAW264.7; cells were kindly provided by Cell Bank, Chinese Academy of Sciences, Shanghai, China) were cultured in cell culture flasks and cultured with complete medium containing 85% Dulbecco's modified eagle medium (high glucose; Gibco, USA) medium, 15% fetal bovine serum (Gibco, USA) and 1% penicillin and streptomycin (Antibiotic‐Antimycotic; Gibco, USA) in an incubator containing 5% CO_2_ humidified atmosphere at 37 °C. The cells passed in a ratio of 1:2 every 3 days.

### Cytokine Secretion Assays

Macrophages were cultured on the surface of SP, SPPL, SPPM, and SPPH with a cell density of 1 × 10^5^ cells per specimen. After incubation for 3 days, the same samples were divided into different groups and irradiated with NIR at different power densities (0, 0.2, 0.5, 0.8 W cm^−2^). Subsequently, the cells were stabilized in a cell incubator for 6 h. Finally, cell supernatants of each group were collected using 1.5 mL EP tubes. Enzyme‐linked immunosorbent assay (ELISA; Anogen) was used to detect TNF‐*α* and TGF‐*β*.

### Real‐Time Polymerase Chain Reaction Analysis

The expression of inflammation‐related genes in macrophages on the surface of samples was quantitatively analyzed by Real‐time polymerase chain reaction (RT‐PCR). Macrophages were cultured on the surface of SP, SPPL, SPPM, and SPPH with a cell density of 1 × 10^5^ cells per specimen and cultured in a cell incubator for 4 days. Furthermore, the SPPM group was selected to investigate the effect of NIR at different power densities (0, 0.2, 0.5, 0.8 W cm^−2^) on macrophages for 5 min. Subsequently, relative quantitative analysis of related gene expression was carried out as described in previous work.^[^ ^74^ ^]^ Specifically, TRIzol Reagent (Invitrogen, Thermo Fisher Scientific Inc., USA) was used for cell lysis to obtain total RNA. Transcriptor first‐strand cDNA Synthesis Kit (Roche, Switzerland) was used to synthesize complementary DNA (cDNA). Glyceraldehyde‐3‐phosphate dehydrogenase (GAPDH) was used as housekeeping gene, and the 2^−ΔΔCt^ analysis method was applied to test the relative expression of target genes.

For in vivo evaluation, the mice were sacrificed by excessive anesthesia and some tissues surrounding implants were stored in liquid nitrogen after 7 and 14 days of implantation. The phagocytic associated genes, TNF‐α, C3, and CD11b genes; inflammation‐related genes, inducible nitric oxide synthase (iNOS), IL‐6, IL‐4, TGF‐β genes; tissue healing associated genes, COL‐I, VEGF, CTGF, α ‐actin (Acta2) genes expression in tissues of each group were also quantitatively analyzed by RT‐PCR. RT‐PCR was carried out by LightCycler 480 System (Roche, Switzerland). All primers were purchased from BioTNT and listed in Table S1, Supporting Information.

### Cytophagocytosis Efficiency

Macrophages were cultured on SP and SPPM samples with a cell density of 1 × 10^5^ cells per specimen for 3 days. Then, the cells cultured on samples were treated with NIR at a power density of 0.5 W cm^−2^ in complete medium for 5 min, and stabilized in a cell incubator at 37 °C for 6 h. The groups without NIR irradiation were denoted as control. Then 5 µg polystyrene microspheres with green fluorescence (PS, Size with 100 nm) were added to each well, gently mixed, and cultured with cells for another 3 h in incubator. Then, the cells cultured on samples were rinsed with PBS to remove the extracellular PS microspheres and digested with 0.05% trypsin for 3 min. The digestion was terminated with complete medium to obtain cells suspension. The cells were cleaned with PBS twice, 5 min each time. Afterward, 4', 6‐diamidino‐2‐phenylindole (DAPI) was used to stain the nuclei in the dark at room temperature for 10 min, and PBS was used to clean again. Finally, the cells were fixed and suspended with 500 µL 4% paraformaldehyde. 50 µL cell suspension was dropped on slide, and the phagocytosis of macrophages in each group was observed under a confocal laser scanning microscope (TCS SP8 SR). And the PS (green fluorescence) was counted around a single nucleus.

### Percutaneous Implantation Infected Model

The experimental protocols employed in this study were approved by the Animal Care and Experiment Committee of Tongren Hospital (2021‐090‐01). 6–8 weeks male C57BL/6J mice (provided by Shanghai Model Organisms Center, Inc.) were selected for the construction of percutaneous implantation model and percutaneous implantation infected model as reported in the literature.^[^ ^10^ ^]^ SP and SPPM cylinders were immersed in *S. aureus* suspension (5 × 10^6^ CFU mL^−1^), and co‐incubated for 1 h. Mice were randomly divided into two groups and anesthetized with 10% chloral hydrate solution. Subsequently, their back hair was removed and the backs were disinfected with iodophor. Kirschner wire (Φ2 mm) was used to create a wound through the skin for inserting SP and SPPM cylinders, both ends of which were fixed with a photocurable adhesive. The groups inserting SP and SPPM cylinders without bacteria were denoted as control. And partial groups with bacteria were irradiated with NIR for 5 min to treat the wound. During the above process, infrared thermal imager was used to measure the temperature changes and infrared thermal images of different implants in mice. Among them, each group of samples contained three conditions: normal group, infected group without or with NIR treatment, which were denoted “‐” and “+,” respectively. After surgery, the mice were kept in a clean room for a specific time.

### Giemsa Staining

After 1 and 4 days of implantation, the tissues around the implants were fixed with 4% paraformaldehyde, dehydrated, embedded, and sectioned. And then they were stained with Giemsa. The slices were scanned by Hamamatsu NanoZoomer S60.

### Western Blot Analysis

C3 protein levels in above tissues were quantified by western blot analysis. In detail, the tissues were lysed by RIPA (A20120A0101, BioTNT) buffer and total protein levels were quantitatively analyzed using a bicinchoninic acid (BCA) kit (1‐PC0020, Solarbio). Then, proteins were resolved by sodium dodecyl sulfate‐polyacrylamide gel electrophoresis (SDS‐PAGE), and transferred to nitrocellulose membranes. Membranes were blocked for 1 h in tris‐buffered saline (TBS) ‐Tween 20 buffer containing 5% (w/v) non‐fat milk, then incubated with primary antibodies against C3 and GAPDH (1:2000; 1: 1000; Abcam, USA) overnight at 4 °C. After that, they were rinsed three times with TBS‐Tween 20 and incubated with horseradish peroxidase‐conjugated secondary antibodies for 1 h at room temperature. As rinsing again, protein bands were visualized using ChemiScope 6100 system (CLiNX, China). The intensity of the protein bands was quantified by Image J software.

### Pathological Analysis

After 7 and 14 days of implantation, partial tissues surrounding implants were fixed with 4% paraformaldehyde, dehydrated, embedded, and sectioned for staining.

Immunohistochemical and immunofluorescence staining passed deparaffinization and rehydration, antigen retrieval, and serum sealing first. Then the slices were incubated with primary antibody overnight at 4 °C. After cleaning with PBS, the secondary antibody was added to incubate for 50 min. DAPI was used to restain the nuclei. For immunohistochemical staining, the primary antibodies, TNF‐*α* (1: 300, GB11188, Servicebio), COL‐I (1: 1000, GB11022‐3, Servicebio), and VEGF (1: 300, GB13034, Servicebio), were used, and HRP labeled secondary antibody (1: 200, GB23303, Servicebio) was applied subsequently. And for immunofluorescence staining, the primary antibodies, F4/80 (1: 50, NB6004045S, NOVUS), CD11b (1: 200, NB110‐894745S, NOVUS), CCR7 (1: 2000, GB11502, Servicebio) and CD206 (1: 1000, GB13438, Servicebio), were used. The second antibodies, FITC labeled goat anti‐rat (1:200, GB22302, Servicebio), CY3 labeled goat anti‐rabbit (1:300, GB21303, Servicebio) and HRP labeled goat anti‐rabbit (1:500, GB23303, Servicebio), were applied. As dyeing was completed, the fluorescence images were observed with Ortho‐Fluorescent microscope (Nikon Eclipse C1, Japan).

In addition, the tissues were also stained with hematoxylin‐eosin (H&E) and Masson to observe. And all images were analyzed by Image‐pro Plus Software (Media Cybernetics Inc., Silver Springs, MD, USA).

### Statistical Analysis

GraphPad Prism7 software was applied for statistical analysis. Quantitative data were expressed as mean ± standard deviation (SD). Statistically significant differences (*P*) were analyzed by *t*‐test, one‐way ANOVA, two‐way ANOVA, and Tukey's multiple comparison tests. A value of *p* < 0.05 was considered statically significant and was represented by the symbol “‘*”’, a value of *p* < 0.01 was represented by “‘**”’, *p* < 0.001 was “‘***”’, and *p* < 0.0001 was “‘****”’.

## Conflict of Interest

The authors declare no conflict of interest.

## Author Contributions

Xi.L. contributed to methodology, formal analysis, and writing—original draft. H.Z. contributed to software and writing‐revision. B.Y. contributed to investigation and writing‐revision. K.W.K.Y. contributed to funding acquisition. Y.L. contributed to funding acquisition and writing—review & editing. L.O. contributed to validation, funding acquisition, and writing—review & editing. Xu.L. contributed to conceptualization, supervision, funding acquisition, and writing—review & editing.

## Supporting information

Supporting Information

## Data Availability

The data that support the findings of this study are available from the corresponding author upon reasonable request.
