# Supplementary material for: On‐Off Phagocytosis and Switchable Macrophage Activation Stimulated with NIR for Infected Percutaneous Tissue Repair of Polypyrrole‐Coated Sulfonated PEEK
Source: Adv Sci (Weinh). 2022 Dec 14;10(5):2205048. doi: 10.1002/advs.202205048 (PMC9929275; doi:10.1002/advs.202205048)
Supplement: Supplementary file 1 — Supporting Information [file ADVS-10-2205048-s001.pdf]

ADVANCED SCIENCE
Open Access

Supporting Information

for *Adv. Sci.*, DOI 10.1002/advs.202205048

On-Off Phagocytosis and Switchable Macrophage Activation Stimulated with NIR for Infected Percutaneous Tissue Repair of Polypyrrole-Coated Sulfonated PEEK

Xingdan Liu, Haifeng Zhang, Bangcheng Yan, Kelvin W. K. Yeung, Yun Liao, Liping Ouyang* and Xuanyong Liu**

On-off phagocytosis and switchable macrophage activation stimulated with NIR for infected percutaneous tissue repair of polypyrrole-coated sulfonated PEEK

Xingdan Liu[1, 2], *Haifeng Zhang*[1, 2, 6], *Bangcheng Yan*[1, 2], *Kelvin W. K. Yeung*[5], *Yun Liao*[3, *], *Liping Ouyang*[3, 4, *], *Xuanyong Liu*[1, 2, 6, *]

[1] State Key Laboratory of High Performance Ceramics and Superfine Microstructure, Shanghai Institute of Ceramics, Chinese Academy of Sciences, Shanghai 200050, China

[2] Center of Materials Science and Optoelectronics Engineering, University of Chinese Academy of Sciences, Beijing 100049, China

[3] Department of Pharmacy, Tongren Hospital, Shanghai Jiao Tong University School of Medicine, Shanghai 200336, China

[4] Hongqiao International Institute of Medicine, Shanghai Jiao Tong University School of Medicine, Shanghai 200336, China

[5] Shenzhen Key Laboratory for Innovative Technology in Orthopaedic Trauma, Guangdong Engineering Technology Research Center for Orthopaedic Trauma Repair, Department of Orthopaedics and Traumatology, The University of Hong Kong Shenzhen Hospital, Shenzhen, 518053, China

[6] School of Chemistry and Materials Science, Hangzhou Institute for Advanced Study, University of Chinese Academy of Sciences, 1 Sub-lane Xiangshan, Hangzhou 310024, China

* Corresponding Author:

Prof. Xuanyong Liu, Dr. Liping Ouyang, and Dr. Yun Liao

Tel.: +86 21 5241 2409. Fax: +86 21 5241 2409.

E-mail: xyliu@mail.sic.ac.cn, lpouyang@shsmu.edu.cn, and libra_ly@shsmu.edu.cn

1. Materials and methods

1.1 Materials characterization

The surface Zeta potential of different samples was detected by the Surpass electrokinetic analyzer (Anton Parr, Austria).

Different samples were treated with ultrasound at 50 KHz and 300 W for 30 min to evaluate the mechanical properties of modified surface. These samples were taken out and dried, and the surface morphologies were observed by field-emission scanning electron microscopy (Magellan 400, FEI, USA).

Adhesive tape method was used to test the adhesion between the coating and substrate. In detail, the samples were adhered to the same tape. Then the tape was removed from the samples, and their pictures before and after tape treatment were taken. Ppy polymerized on PEEK surface, which was denoted as control. Image J software was used to calculate the residual coating percentage on the substrate surface.

1.2 Macrophages response *in vitro*

Macrophages were seed on different samples (four replicates) with a density of 1×10^5 and cultured in 24-well plates for 4 hours, 1 day and 4 days. AlamarBlue^{TM} (Thermo Fisher Scientific Inc., USA) assay was used to evaluate cell viability. At every point in time, 500 μL fresh full culture medium with 10% alamarBlue^{TM} was added into each well and incubated for 2 hours sequentially in dark. Then 100 μL solution was taken out to detect its fluorescence intensity at excitation wavelength of 560 nm and emission wavelength of 590 nm by the microplate reader (Cytation5,

USA). Additionally, the samples with cells were taken out and fixed with 2.5% glutaraldehyde solution in the dark overnight. The cells cultured on samples surface were dehydrated for 10 min through a series of ethanol solutions (30, 50, 75, 90, 95 and 100% v/v) and the hexamethyldisilazane-ethanol solutions (1:2, 1:1 and 2:1 v/v) sequentially. Finally, cells morphologies were observed with scanning electron microscopy (S-3400N TypeI, Hitachi, Japan).

The inflammation-related genes expression in macrophages was evaluated by RT-PCR. The cells were seed on different samples (four replicates) with a density of 1×10^5 in 24-well plates for 4 days. And the gene expression levels of TNF-α, IL-6, IL-10 and IL-4 were detected as described in section "Real-Time Polymerase Chain Reaction Analysis".

The flow cytometry was used to detect macrophages polarization on SP and SPPM samples under different power densities of NIR. Macrophages were seed on samples with a density of 1×10^5 and cultured in 24-well plates for 3 days. And then, the cells cultured on samples (four replicates) surface were treated with 0, 0.2, 0.5 and 0.8 W/cm^2 NIR, respectively. Sequentially, they continued to be cultured for 6 hours. After that, the cells were digested with 0.05% Ethylene diamine tetraacetic acid (EDTA)-trypsin and incubated with rat anti-mouse CD16/CD32 antibody (BD Pharmingen, USA) to block Fc-receptors. After washed and resuspended with PBS, the cells were incubated with phycoerythrin (PE)-conjugated anti-mouse F4/80 antibody (BD Pharmingen, USA), PE/Cy7-labeled anti-mouse CD197 (CCR7) antibody (Biolegend, USA) and allophycocyanin (APC)-conjugeted anti-mouse

CD206 antibody (BD Pharmingen, USA) for 30 min. After washed again, the cells were transferred into fluorescence activated cell sorting (FACS) tubes and a flow cytometer (BD, Celesta) was used to detect 20,000 events per tube.

1.3 Fibroblasts response *in vitro*

Mouse fibroblast cell line (L929, provided by Cell Bank of Chinese Academy of Sciences, Shanghai, China) was also used to evaluate the cytocompatibility. L929 cells were seed on different samples with a density of 2×10^4. After culturing for 1, 4 and 24 hours, the cells were fixed with 4% paraformaldehyde overnight at 4 °C. Then they were permeabilized with 0.1% (v/v) Triton X-100 for 2 min and 1% BSA were added to block for 30 min. Finally, cells were stained with fluorescein isothiocyanate (FITC)-phalloidin (Sigma, USA) for 1 hour and stained with DAPI for another 10 min. The cytoskeleton was observed by a confocal laser-scanning microscope (CLSM, LeicaSP8, Germany).

L929 cells with a density of 2×10^4 per well were seed on various samples (four replicates) and cultured for 1, 4 and 7 days. After each incubation period, cells proliferation and morphologies were detected as described in section "Macrophages response *in vitro*".

The tissue healing related genes expression in L929 cells was evaluated by RT-PCR. The cells were seed on different samples (four replicates) with a density of 1×10^5 in 24-well plates for 7 days. And the gene expression levels of *COL-I*, *TGF-β1*, *CTGF* and *Acta 2* were detected as described in section "Real-Time Polymerase Chain Reaction Analysis".

1.4 Toxicity assessment in percutaneous implantation model

Different groups were implanted in the back of mice. After 14 days, the major organs of mice were collected. And the biosafety was evaluated by H&E staining of heart, liver, spleen, lung, and kidney. The untreated group was as control.

2. Results

2.1 Surface properties evaluation

At pH=7.4, the surface zeta potential gradually shifted positively with the increase of Ppy content on SP (Figure S1). Figure S2 showed surface morphologies of different samples after ultrasound for 30min. The porous structure on SP samples surface collapsed, while the porous morphologies on SPPM and SPPH samples surface retained due to the package of Ppy particles. And the structural damage degree of SPPL samples was between SP and SPPM samples.

To test the adhesion between Ppy and the substrate, adhesive tape method was used, and the results were shown in Figure S3. The PEEK and SP samples coating with Ppy both showed dark surface. After application with tape methods, the area of remaining Ppy on PEEK and SP samples were 32.5% and 93% respectively.

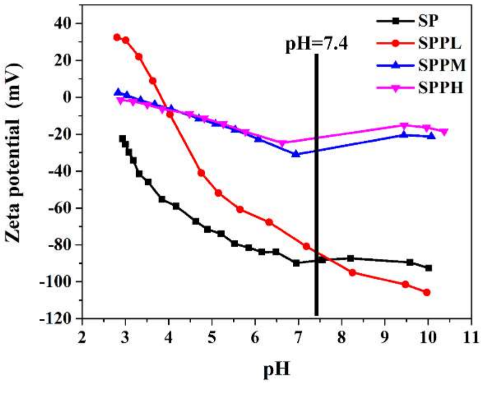

Figure S1. Zeta potential of different samples.

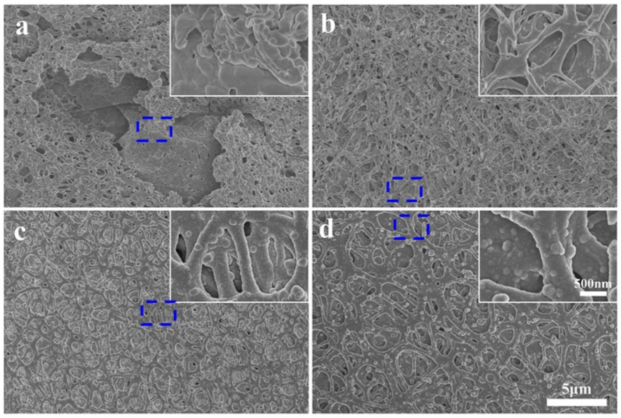

Figure S2. Surface morphologies images of different samples after 30min of ultrasound at 50 KHz and 300 W. (a) SP; (b) SPPL; (c) SPPM; (d) SPPH. The insert on top right corner is high magnification of dash line marked area, respectively.

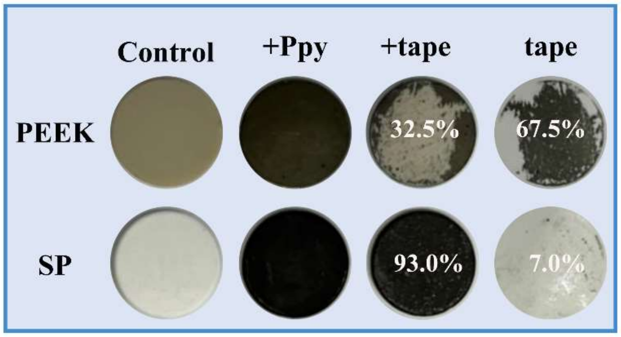

Figure S3. Macro-images of Ppy modified PEEK and sulfonated PEEK samples processed by adhesive tape.

2.2 Antibacterial abilities

Figure S4-5 showed colony plate images for *S. aureus* cultured on SPPM samples surface under different treatment times of NIR. The results were consistent with those in Figure 3c-d. It could be seen that 0.5 W/cm^2 NIR stimulation within 30 s in air and within 5 min in aqueous solution could achieve more than 80% bacteriostasis rate. Figure S6 showed the anti-*S. aureus* abilities of SPPL and SPPH samples under 0.5

W/cm^2 NIR for 5 min. After NIR irradiation, both SPPL and SPPH groups had certain bacteriostasis. The bacteriostasis rate of SPPH group with NIR was more than 99%.

Figure S7-8 showed colony plate images for *E. coli* cultured on SPPM samples surface under different treatment times of NIR. And 0.5 W/cm^2 NIR stimulation within 60 s in air and within 5 min in aqueous solution could achieve more than 85% bacteriostasis rate, which was consistent with those in Figure 3i-j. Figure S9 showed the anti-*E. coli* abilities of SPPL and SPPH samples under NIR for 5 min. Both SPPL and SPPH groups had certain bacteriostasis as NIR treatment. The bacteriostasis rate of SPPH group with NIR was more than 99%.

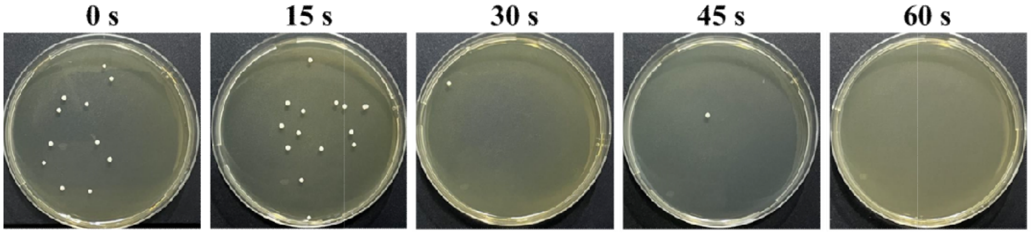

Figure S4. Bacterial colony plate images at 200 times dilution of SPPM group inoculated with *S. aureus* after irradiation at 0.5 W/cm^2 NIR in air for different times.

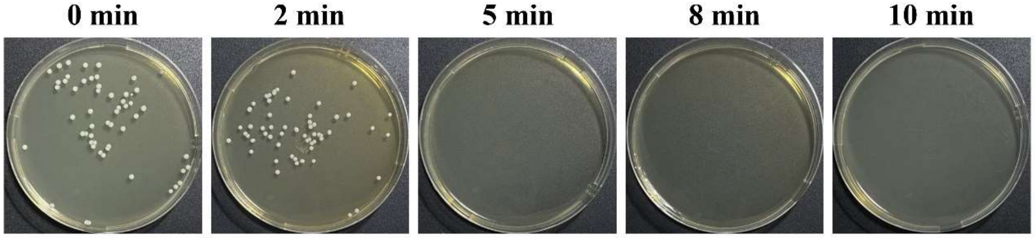

Figure S5. Bacterial colony plate images at 200 times dilution of SPPM group inoculated with *S. aureus* after irradiation at 0.5 W/cm^2 NIR in physiological saline for different times.

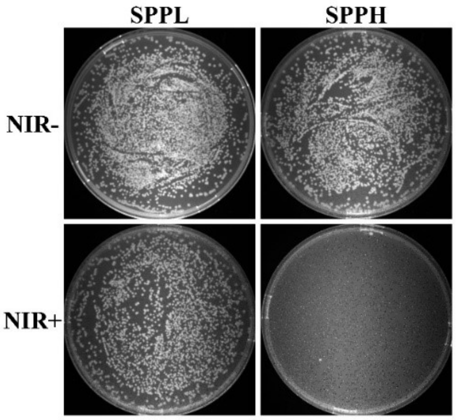

Figure S6. Bacterial colony plate images without dilution of *S. aureus* on SPPL and SPPH samples surface irradiated with 0.5 W/cm^2 NIR for 5 min.

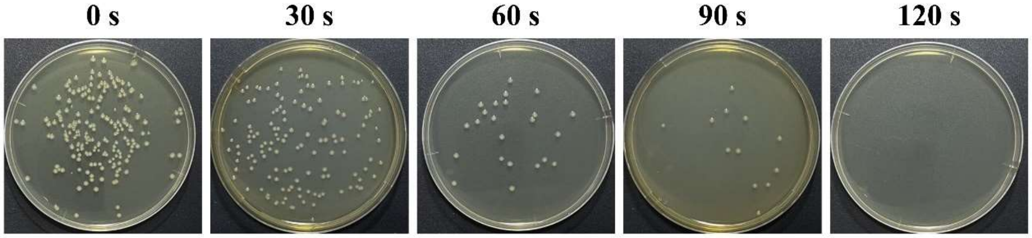

Figure S7. Bacterial colony plate images at 1000 times dilution of SPPM group inoculated with *E. coli* after irradiation at 0.5 W/cm^2 NIR in air for different times.

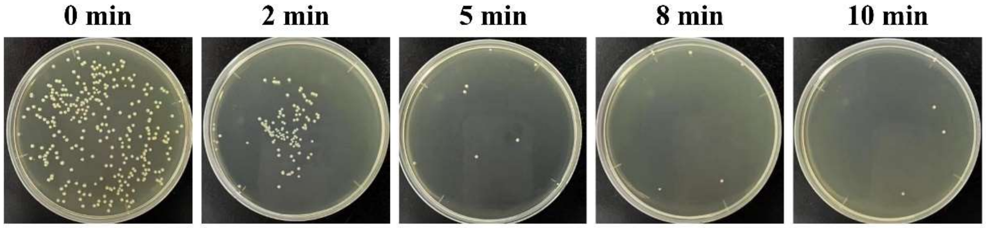

Figure S8. Bacterial colony plate images at 2000 times dilution of SPPM group inoculated with *E. coli* after irradiation at 0.5 W/cm^2 NIR in physiological saline for different times.

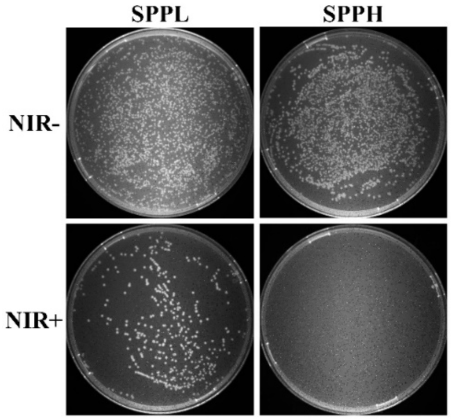

Figure S9. Bacterial colony plate images at 100 times dilution of *E. coli* on SPPL and SPPH samples surface irradiated with 0.5 W/cm^2 NIR for 5 min.

2.3 Macrophages response *in vitro*

The proliferation of macrophages cultured on the samples surface at the later culture time were enhanced compared with that at the previous culture time, which indicated that all samples showed well biocompatibility to macrophages (Figure S10). And macrophages cultured on Ppy modified samples surface had more pseudopodia and spread area than those cultured on SP samples with spherical shape (Figure S11).

Figure S12 showed the expression of inflammation-related genes in macrophages cultured on different samples. SPPM group significantly inhibited *TNF-α* and *IL-6* genes expression compared with SPPL and SPPH groups. And there was no significant difference in *TNF-α* and *IL-6* genes expression regulation between SPPM and SP groups. Furthermore, SPPM group significantly up-regulated the expression of *IL-10* gene compared with the other three groups. In addition, there was no significant difference between SP and SPPL groups, while SPPH group substantially enhanced these genes expression compared with the SP group for *TNF-α*, *IL-6*, *IL-10*, and *IL-4*.

Figure S13 showed the effect on macrophages phenotype cultured on samples under different power densities of NIR. With increased power density of NIR, M1/M2 increased and there had highest M1-type macrophage proportion under 0.5 W/cm^2 in SPPM group. However, there was no significant effect on the proportion of M1-type macrophages, and it only regulated the proportion between unpolarized and M2-type macrophages in SP group.

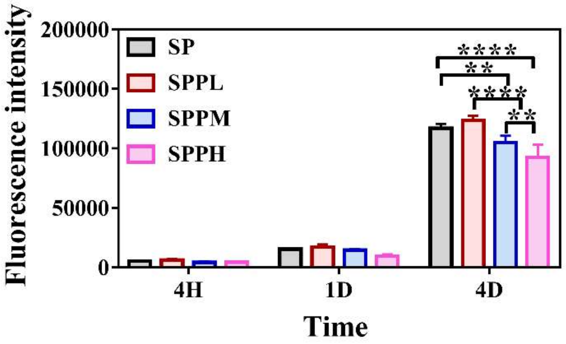

Figure S10. Proliferation of macrophages cultured on different samples. Data represent means ± SD (n = 4). Statistical significance was calculated by two-way ANOVA analysis and Tukey's multiple comparison tests. *p < 0.05; **p < 0.01; ***p < 0.001; ****p < 0.0001.

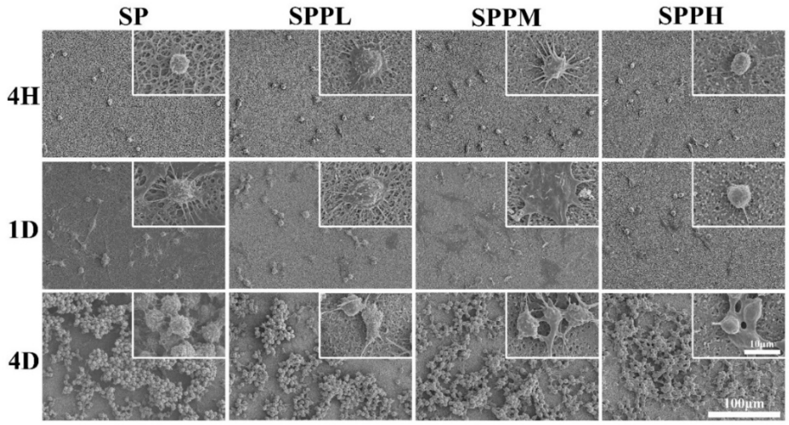

Figure S11. Morphologies of macrophages cultured on different samples.

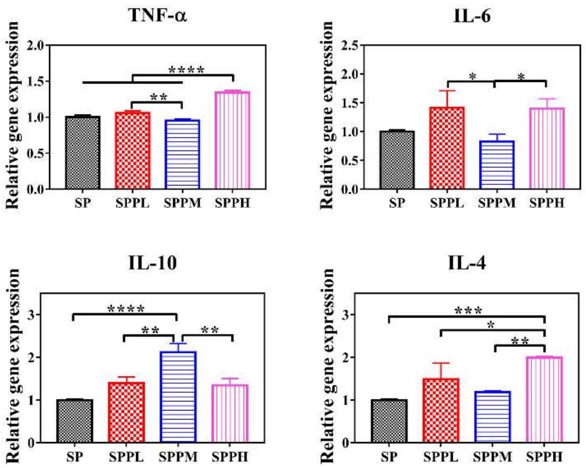

Figure S12. Expression of inflammation-related genes in macrophages cultured on different samples. Data represent means ± SD (n = 3). Statistical significance was calculated by one-way ANOVA analysis. *$p < 0.05$; **$p < 0.01$; ***$p < 0.001$; ****$p < 0.0001$.

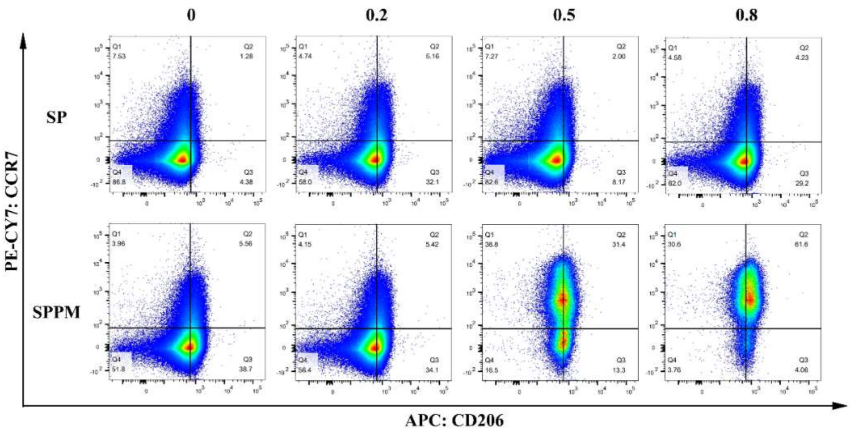

Figure. S13. Flow cytometry of macrophage phenotypes cultured on SP and SPPM samples after NIR stimulation at different power densities (Unit: W/cm^2) for 5 min (M1: CCR7; M2: CD206).

2.4 Macrophages polarization in percutaneous implantation infected model

M1-type (CCR7: green), M2-type (CD206: red) and nucleus of macrophages in tissue

were marked in Figure S14. After implantation for 7 days, M1/M2 proportion in SPPM groups was higher than that in SP groups while its proportion in NIR group was also higher than that in non-NIR group. After 14 days, M1/M2 proportion in each group was decreased compared with that at 7 days, and SPPM group with NIR had lowest M1/M2 proportion than the other groups with no significant difference.

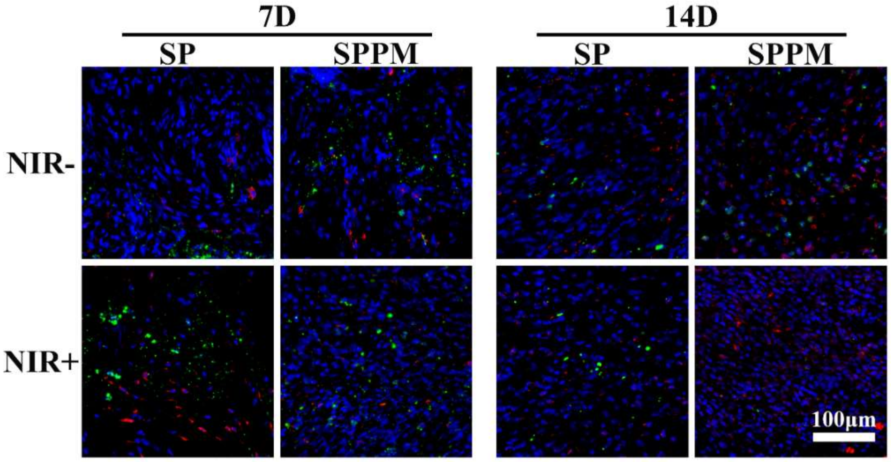

Figure S14. Immunofluorescence staining images of macrophages phenotype marker for surrounding tissue of SP and SPPM groups in the percutaneous implantation infected model at 7 and 14 days. M1: CCR7: green, M2: CD206: red and nucleus: Blue.

2.5 Biosafety evaluation

The major organs of mice were stained by H&E after implantation for 14 days in the percutaneous implantation model. All the organs in experimental group exhibited normal states, which suggested the good biosafety of SP and SPPM samples *in vivo* (Figure S15).

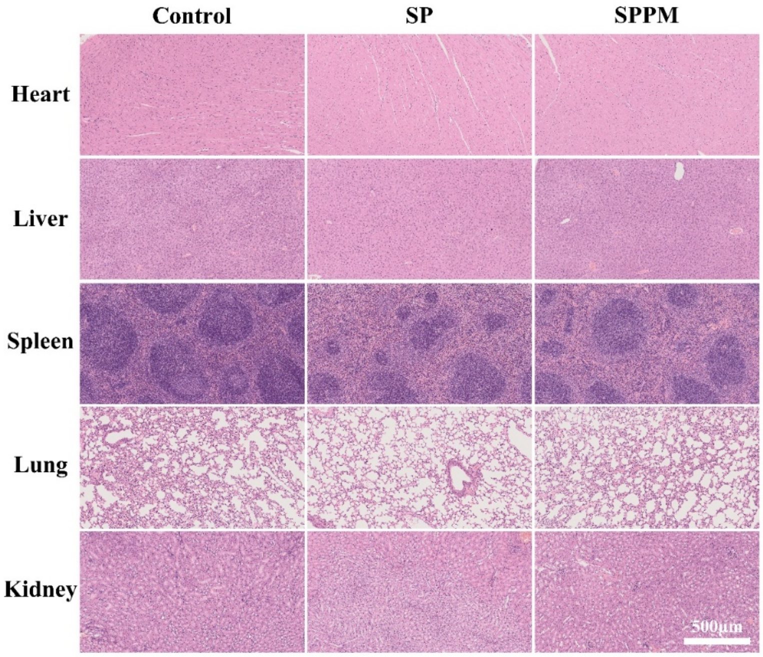

Figure S15. H&E staining images of major organs from SP and SPPM groups in the percutaneous implantation model at 14 days.

2.6 L929 response *in vitro*

The cytoskeleton of L929 in different groups was observed in Figure S16. Cells in each group were spread out as incubation time extended. L929 on Ppy modified samples had larger spread area than that on SP samples. Figure S17 and Figure S18 showed proliferation and morphologies of L929, respectively. Cells in Each group had good activity, and they could form biofilms after incubation for 7 days. L929 in SPPH group showed marked stretching compared with the other groups.

The expression of tissue healing related genes in L929 was tested by RT-PCR and results were shown in Figure S19. For *COL-I* and *TGF-β1* genes, Ppy modified group could up-regulate their expression significantly compared with SP group. With the

increased Ppy content on surface, it could promote *COL-I* and *TGF-β1* genes expression while there was no significant difference between SPPM and SPPH groups. For *CTGF* gene, SPPL group down-regulated while SPPM and SPPH groups up-regulated its expression compared with SP group. The *CTGF* gene expression was highest in SPPM group. Ppy modified group with no significant difference down-regulated *Acta 2* gene expression compared with SP group.

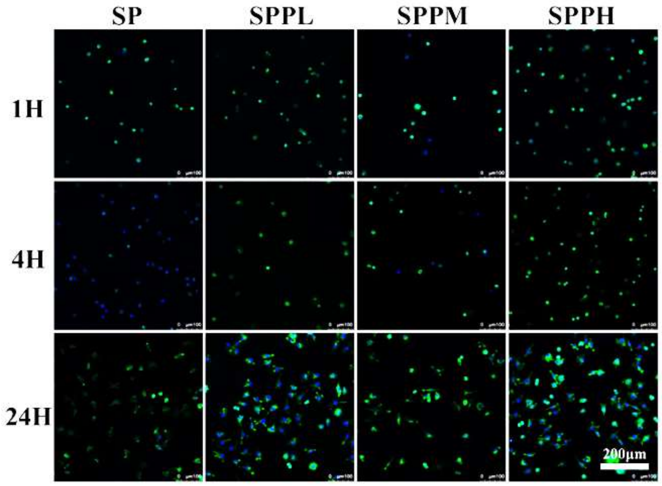

Figure S16. Cytoskeleton of L929 cultured on different samples at 1, 4 and 24 hours.

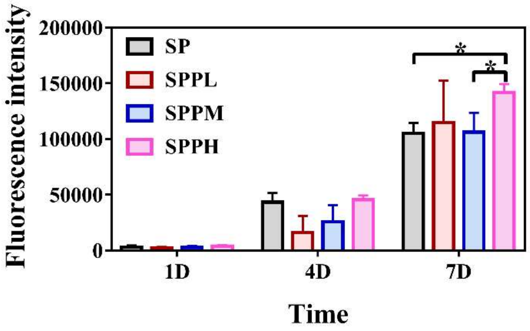

Figure S17. Proliferation of L929 cultured on different samples. Data represent means ± SD (n = 4). Statistical significance was calculated by two-way ANOVA analysis and Tukey's multiple comparison tests. *$p < 0.05$; **$p < 0.01$; ***$p < 0.001$; ****$p < 0.0001$.

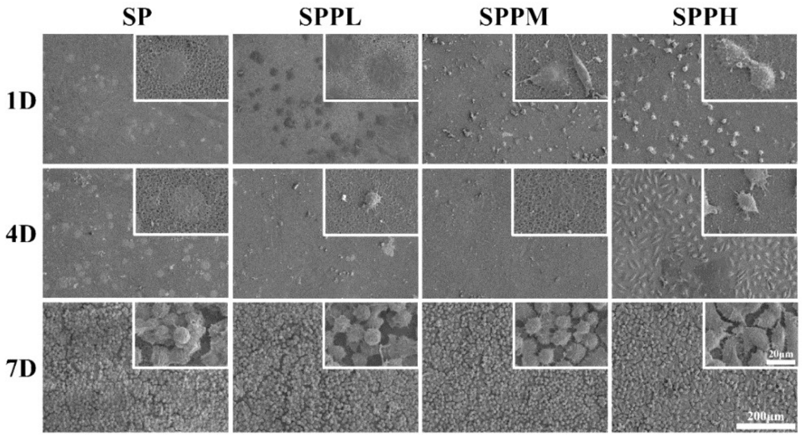

Figure S18. Morphologies of L929 cultured on different samples.

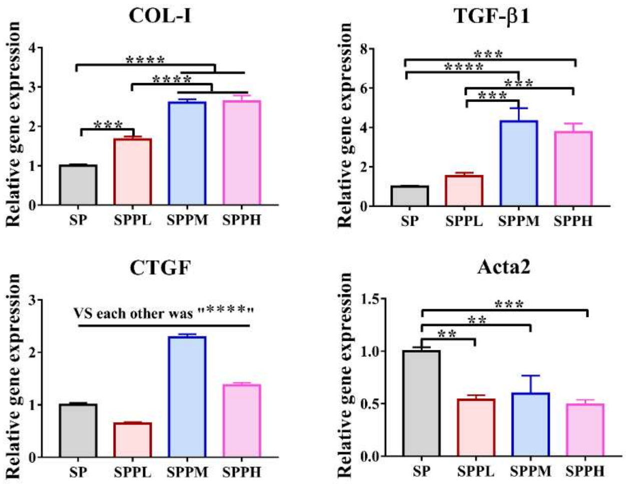

Figure S19. Expression of tissue healing related genes in L929 cells cultured on different samples. Data represent means ± SD (n = 3). Statistical significance was calculated by one-way ANOVA analysis. *p < 0.05; **p < 0.01; ***p < 0.001; ****p < 0.0001.

Table S1 Primers of genes used in the RT-PCR experiment.

Gene (mouse)	Primer sequences (F, forward; R, reverse; 5'-3')	Product size (bp)

GAPDH	F: GCT CAG GCC TCT GCG CCC T<br>R: CCT ACT CTC TTG AAT ACC	115
TNF-α	F: TTA GAG CGG GAT AGT AAC G<br>R: CAA AAT ACA CAA CAG TGT C	111
IL-6	F: CCA AGA GGT AAA AGA TTT AC<br>R: ATT GAT AAT TTA AAT AAG TA	161
IL-10	F: CCC TTT GCT ATG GTG TCC T<br>R: GTG GCC AGT TTG TTA TTT AT	106
IL-4	F: CCA TGA ATG AGT CCA AGT CC<br>R: TAA CTT ATG AAT TTT TAA T	60
TLR4	F: CAC TAA CGG GAG AAT CCT GTG<br>R: AGG GAC CAT CTT CAT TTC CAT	118
MyD88	F: AGC AGT GTC CCA CAA ACA AAG<br>R: GGC AGT AGC AGA TAA AGG CAT	100
TRAF6	F: ACT GCT GAG TGT TAC TGC CAT<br>R: GCT GTC ATC ATC CAC GAG A	163
NF-κB1	F: TGA CAA GGT TCA GAA AGA TG<br>R: GAA GAC AAT GGC AAA CTG	124
C3	F: AAC AAG AAC ACC CTC ATC ATC<br>R: GCT GGA TAA GTC CCA CAT TAA	103
CD11b	F: CGG ATT CAC TTC ACC TTC AAT<br>R: CCC TGA GGC AGT TTT TGT C	102
iNOS	F: TTG ACG CTC GGA ACT GTA<br>R: GTT GGT GGC ATA AAG TAT GTG	74
TGF-β	F: AAG GAC CTG GGT TGG AAG T<br>R: GGT CCT TGC CCT CTA CAA C	135
TGF-β1	F: AAC CAA GGA GAC GGA ATA CA<br>R: CGT GGA GTT TGT TAT CTT TGC	76
CTGF	F: CTC TGA CCA TTC TGA TTC CA<br>R: GGC TTG TTA CAG GCA AAC TTA C	97
Acta 2	F: CTC TGC CTC TAG CAC ACA ACT<br>R: CCA CGA GTA ACA AAT CAA AGC	98
COL-I	F: TGA CTG GAA GAG CGG AGA GTA<br>R: GAC GGC TGA GTA GGG AAC AC	117
VEGF	F: GAT CAG ACC ATT GAA ACC AC<br>R: GAA GAT GAG GAA GGG TAA GC	79
